# Cerium Dimer Anion and the Contribution of 4f Electrons to Lanthanide Metal–Metal Bonds

**DOI:** 10.1021/jacs.5c07348

**Published:** 2025-09-25

**Authors:** Jiaye Jin, Tatsuya Chiba, Max Grellmann, Shiying Wang, Marcel Jorewitz, Kit H. Bowen, Roland Mitric, Knut R. Asmis

**Affiliations:** † Wilhelm-Ostwald-Institut für Physikalische und Theoretische Chemie, 9180 Universität Leipzig, Linnestr. 2, 04103 Leipzig, Germany; ‡ Institut für Physikalische und Theoretische Chemie, Universität Würzburg, Emil-Fischer Str. 42, 97074 Würzburg, Germany; § Department of Chemistry, 1466 Johns Hopkins University, Baltimore, Maryland 21218, United States

## Abstract

Direct metal–metal bonding between lanthanide atoms has been challenging to observe. We report on the first spectroscopic characterization of the cerium dimer anion (Ce_2_^–^) and its neutral analog (Ce_2_) in the gas phase, achieved using photoelectron and ultrafast spectroscopy combined with high-level quantum chemistry calculations. The electron affinity of Ce_2_ is 0.24 eV, from which a dissociation energy of 2.21 eV is derived for Ce_2_^–^. The wave-packet dynamics upon photodetachment are studied and yield vibrational frequencies for electronically excited Ce_2_. Ce_2_^–^ exhibits a conventional metal–metal triple bond with minimal contribution from 4f electrons. However, evidence of 4f-electron participation in bonding is identified for the low-energy excited states only 0.1 eV higher. The results challenge the assumption of inert 4f electrons in metal–metal bonding, and we propose a promising strategy for forming stable lanthanide–lanthanide bonds involving significant 4f-electron contributions.

## Graphical abstract

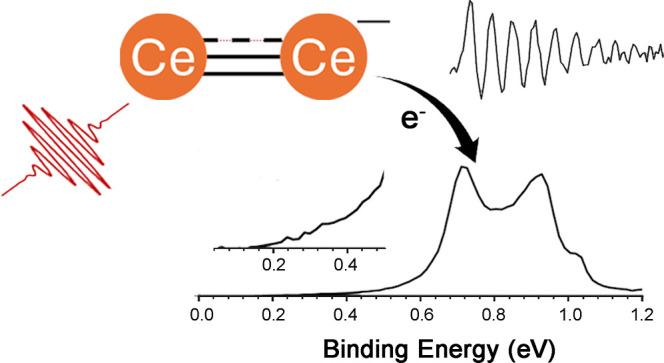

## Introduction

Direct metal–metal interactions in chemical compounds exhibit unique electronic properties and reactivity, that can be exploited for advancing catalysis and improving functional materials.^1–3^ In particular, the f-elements (lanthanides and actinides), with their valence f orbitals, exhibit greater complexity that allows for the expansion of applications by enhancing optical and magnetic properties.^4,5^ Actinides with occupied 5f orbitals stand out for engaging in covalent metal–metal bonding under mild conditions.^6,7^ A notable example is the uranium dimer,^8^ where direct 5f-5f interactions stabilize both the neutral dimer U_2_ and its anion U_2_^–^ (see Scheme 1). Conversely, the participation of 4f orbitals in chemical bonding remains rare. Typically, it is limited to extreme conditions such as high pressure,^9^ as well as to specific oxides and halides of light lanthanides.^10–12^ This inertness stems from the highly localized nature of 4f orbitals, whose contracted radial distribution leads to negligible bonding contributions in most cases. Evidence of 4f-orbtial covalency in metal–ligand bonds has been recently observed in the lanthanides complexes with strong 4f/5d hybridization.^13–16^ Observation of direct metal–metal bonding between lanthanide atoms and understanding the covalent 4f contributions therefore represents a long-standing goal in inorganic chemistry and remains a challenge.

**Scheme 1:**
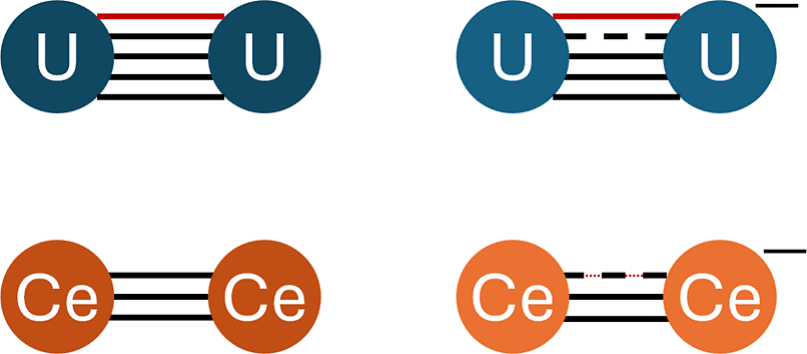
Chemical Bonding of U_2_^0/–^ and Ce_2_^0/–^

As the first lanthanide element with an occupied 4f orbital in its ground state ([Xe]­4f^1^5d^1^6s^2^), Ce has the largest ionic radius in the 4f series, making it a unique case for examining 4f-electron contributions to bonding and catalysis.^14,15,17^ Isolated metal clusters in the gas phase represent ideal model systems for studying metal–metal bonding, using the highly sensitive and selective tool kit of ion spectroscopy combined with highest-level theoretical approaches.^18–24^ This provides fundamental insight into metal–metal interactions in the absence of environmental perturbations, allows benchmarking of computational methods and lays the foundations for the rational design of compounds with tailored properties.

The cerium dimer, Ce_2_, represents the simplest system for a direct investigation of Ce–Ce bonding, yet surprisingly few spectroscopic and theoretical studies have explored its nature, in particular with respect to the role of 4f-electron contributions to homonuclear covalent bonding.^25–30^ While Ce_2_ has been detected using photoionization mass spectrometry,^30^ it has not been investigated spectroscopically in the gas phase. A single Raman spectroscopy of Ce_2_ in a solid argon matrix provided vibrational information.^25^ Subsequently, refined theoretical studies suggest no direct 4f contribution to metal–metal bonding, predicting a Ce–Ce triple bond primarily from 5d and 6s orbitals.^26,27^ Intriguingly, a key aspect unaddressed by previous studies is the electron affinity (EA) of Ce_2_, one of its fundamental properties, which explores how electron attachment might alter its electronic structure and bonding, especially regarding 4f-orbital participation in the anion. Indeed, both spectroscopic and theoretical data on Ce_2_^–^ remain largely unexplored. The challenges associated with its characterization stem from the strong reducing properties of Ce, which complicate the gas-phase production, and its highly complex electronic structure–an issue already encountered in previous studies on the atomic anion, Ce^–^.^31–36^ Here, we present the first spectroscopic investigation of Ce_2_^–^ and Ce_2_ in the gas phase, using photoelectron and ultrafast spectroscopy, supported by high-level quantum chemistry calculations. Our work reports the EA of Ce_2_, and highlights the previously unexplored Ce_2_^–^, revealing how an additional electron enhances the 4f-electron contribution to Ce–Ce bonding.

## Results and Discussion

### Mass Spectrometry

Cerium dimer anions, Ce_2_^–^, were generated in the gas phase by aggregation, using a cerium target as the anode of a DC magnetron sputtering source kept at 130 K, as described previously.^37,38^ The cerium target was initially sputtered for several hours to clean surface by removing contaminated layers. Moreover, collisions were kept at a minimum through the short aggregation distance and a low gas flow, to avoid excessive collisions with residual gaseous molecules in the vacuum chamber. A typical quadrupole mass spectrum in the *m*/*z* range between 100 and 360 (see Figure 1A) shows the monomer anion ^140^Ce^–^ as the most abundant anion and the dimer anion ^280^Ce_2_^–^ with a relative abundance of 0.6% with respect to the monomer. The magnified section shows the two most abundant natural isotopologues of Ce_2_^–^, ^280^Ce_2_^–^ and ^282^Ce_2_^–^, confirming the observation. Under these sputtering conditions, the formation of other anions, e.g. oxides or hydrates, are negligible, in contrast to previously reported mass spectra.^39^ Ce_2_^–^ was also generated by laser ablation of a cerium rod using a pulse of helium gas (see Figure 1B for a typical time-of-flight mass spectrum). Besides Ce_2_^–^, other anions containing oxygen, hydrogen, and carbon atoms are observed as well, probably due to the residual gas in the gas line, the surface oxide/hydride on the cerium rod, and hydrocarbons in the stainless-steel housing of the laser ablation setup. Remarkably, Ce_2_H^–^ is about 1.2 times more intense than Ce_2_^–^.

**Figure 1:**
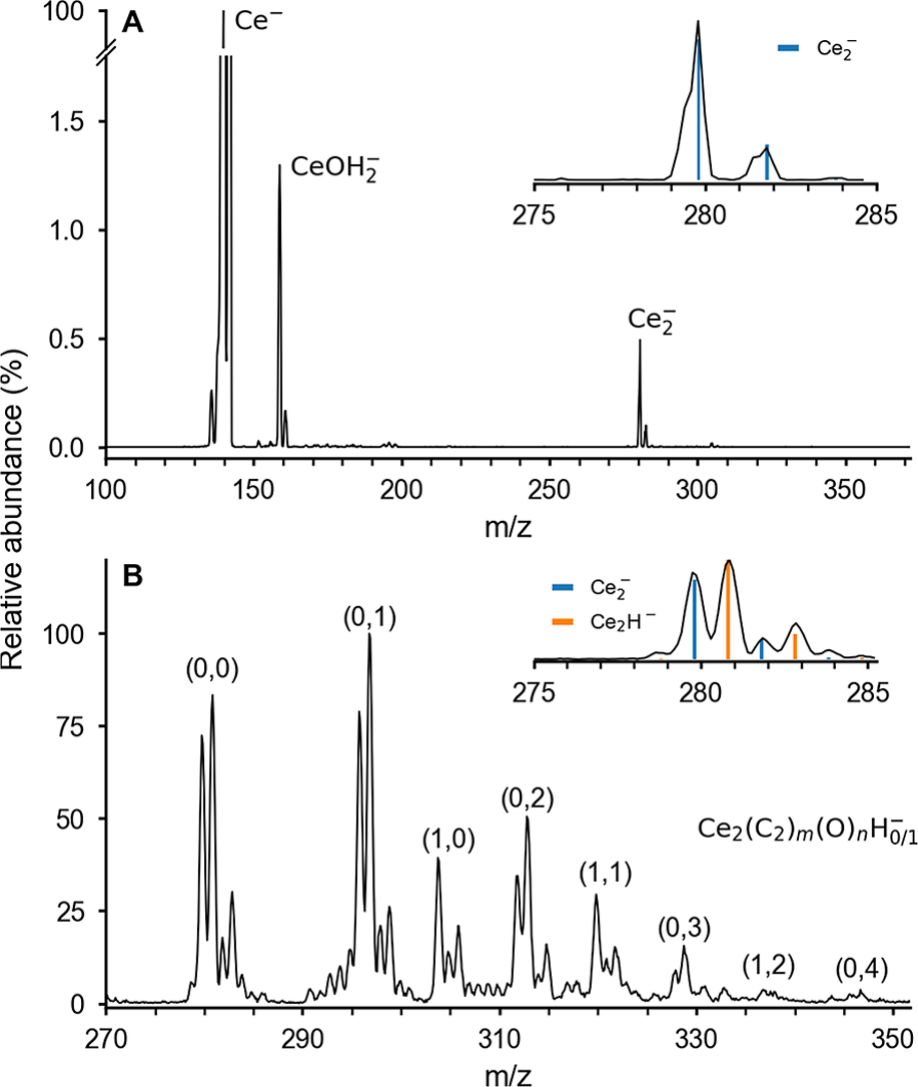
Typical mass spectra obtained by (A) sputtering and (B) laser ablation of a cerium target. The magnified spectra are shown for the Ce_2_^–^ isotopologues. Natural abundance is plotted in bars.

### Anion Photoelectron Spectroscopy

The anion photoelectron (APE) spectrum of Ce_2_^–^ was recorded at a photon energy of 1.17 eV (1064 nm) using a magnetic bottle (MB) electron energy analyzer^40^ and a velocity-map imaging (VMI) electron energy analyzer^41^ to investigate its electron binding energy (eBE). The spectrum taken by the MB analyzer (Figure 2) shows two prominent peaks at 0.71 and 0.93 eV with a shoulder feature appearing at 1.02 eV, which are also observed in the spectrum taken by VMI (Figure S1). Although the VMI analyzer has a slightly better resolution (Δ*E*/*E* ≈ 0.03) than the MB analyzer (0.035 at 1 eV), its lower collection efficiency results in a reduced signal-to-noise ratio. The photoelectrons of these two peaks show anisotropic distributions aligned with the polarization direction of the photodetachment laser (see the reconstructed VMI image in Figure S1B). The low eBE region of the APE spectrum reveals the information about the origin transition corresponding to the EA. The magnified view inserted in Figure 2 reveals the lowest eBE band at 0.24 eV, from which the EA­(Ce_2_) is determined, with two additional peaks at 0.28 and 0.33 eV. The tail below EA, onsetting at 0.15 eV, is attributed to unresolved “hot bands” associated with Ce_2_^–^. Additional MB APE spectra of Ce_2_^–^ (Figures S2 and S3) were also recorded using higher photon energies of 2.33 eV (532 nm) and 3.49 eV (355 nm). Both spectra show a dominant peak at 1.7 eV and weak features below 1 eV. The dissociation energy *D*_0_(Ce_2_^–^) can be determined using the relation *D*_0_(Ce_2_) + EA­(Ce_2_) = *D*_0_(Ce_2_^–^) + EA­(Ce). This yields 2.21 eV for *D*_0_(Ce_2_^–^), based on the measured EA­(Ce_2_) of 0.24 eV, the known EA­(Ce) of 0.60 eV,^31^ and the reported *D*_0_(Ce_2_) of 2.57 eV in a solid argon matrix.^25^

**Figure 2:**
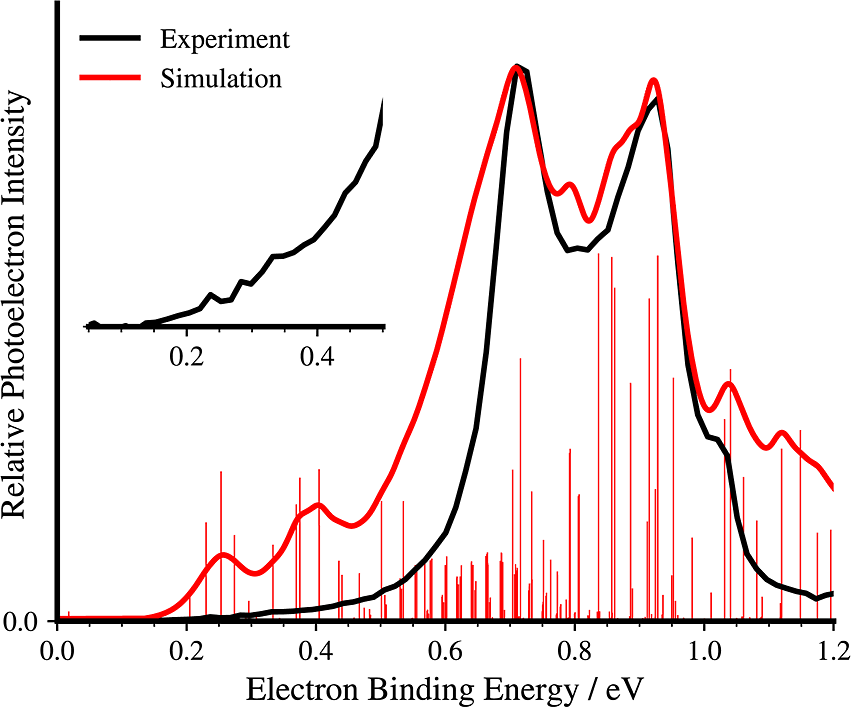
Experimental anion photoelectron spectrum of Ce_2_^–^ obtained with 1.17 eV photon energy (black) compared to the simulated spectrum derived from SO-CASSCF calculations (red). Red sticks: transition energies and intensities calculated at 50 K, red curve: convoluted spectra using a Lorentzian line shape with 0.055 eV width and shifted by −0.24 eV (see text). The inset shows the low eBE region of the experimental spectrum, highlighting the onset of the signal.

The photodepletion (PD) spectrum of Ce_2_^–^ was measured as a function of the photon energy from 0.50 to 0.80 eV, by monitoring the Ce_2_^–^ counts after irradiation with a tunable laser, as shown in Figure S8. The PD spectrum of Ce_2_^–^ aligns well with the features in the APE spectrum. The main band at 0.72 eV is consistent with the onset of the dominant band centered at 0.71 eV in the APE spectrum. The reason for the higher cross section at higher photon energy is not clear yet. Probably the population of electronically excited states of Ce_2_^–^ leads to efficient photodetachment or photodissociation. Note, excited electronic states above the photodetachment threshold have recently been observed in anionic silver clusters using a similar approach.^42^

The APE spectrum is compared to a simulated spectrum (given in red in Figure 2) derived from calculations using the spin–orbit complete active space self-consistent field (SO-CASSCF) method with a large-core effective core potential (ECP). This approach allows access to the high-lying electronic states responsible for the intense experimental features in the region of interest. Details on the electronic structure calculations and spectrum simulation are provided in the Supporting Information. The calculated potential energy surfaces (PESs) of electronic states are given in Figures S4 and S5. The spectrum was simulated by convoluting the stick spectrum obtained from a Franck–Condon analysis involving the transitions between the vibronic states of Ce_2_^–^ and Ce_2_ at 50 K. A detailed description of the spectra simulations is given in the Supporting Information. The selected temperature reflects the typical conditions of gas-phase experiments. See Figures S6 and S7 for the decomposition of electronic state densities and simulated spectra at different temperatures. The simulated spectrum is shifted by −0.24 eV to align with the two most intense bands observed at 0.71 and 0.93 eV for a better comparison with the experimental spectrum. Such discrepancies between calculated electronic energies and experimental eBEs are frequently encountered in the electronic spectra of clusters involving metal–metal bonding.^18,43,44^ The simulation reproduces the most prominent spectral features well, including the gradual onset of low-energy signals and a high-energy shoulder near 1.0 eV. At low photon energies, electron detachment primarily occurs from the nonbonding 6σ_u_ orbital, generating the low-energy shoulder between 0.2 and 0.4 eV. Notably, the intensity of the bands in the APE spectrum at lower energies is not fully reproduced by the Franck–Condon analysis, likely due to a more comprehensive photodetachment mechanism resulting from the dependence of the electron emission cross section on the photon energy, as discussed previously.^45–47^ The calculations predict a significant increase in the density of electronic states in Ce_2_ between 0.4 and 1.0 eV, contributing to the formation of the prominent spectral features at 0.71 and 0.93 eV, where multiple electronic transitions become accessible (Tables S1–S4). These two dominant bands corresponding to the transitions to electronic states in Ce_2_ result from electron detachment from the bonding 5π_u_ orbitals of Ce_2_^–^ (Table S1). The calculated EA is determined as the adiabatic energy between the ground states of Ce_2_^–^ and Ce_2_, yielding a value of 0.11 eV within our SO-CASSCF approach, slightly lower than the experimental EA, 0.24 eV. As expected, the simplified treatment of the 4f electrons results in a less accurate description of the electronic transitions.

### Femtosecond Pump–Probe Spectroscopy

Vibrational information upon photoexcitation offers valuable insights into the bond strength associated with electronic states and also serves to validate our quantum chemistry calculations. We therefore applied femtosecond (fs) pump–probe spectroscopy using the “negative ion—to neutral—to positive ion” (NeNePo) excitation scheme,^37,38,48^ to track the vibrational wave-packet dynamics of Ce_2_ following the photodetachment within fs temporal resolution (see Supporting Information for details). Given the nature of quantum beats, the beating frequencies obtained from the oscillatory transients not only reveal the energy differences between vibrational levels, but also aid in disentangling highly dense electronic states observed in the APE spectrum. We focus on excitation energies from 0.52 to 0.86 eV, corresponding to the most prominent features. Note, our current laser wavelength is limited to the excitation energies higher than 0.5 eV (2500 nm), impeding probing the ground electronic state of Ce_2_ near 0.24 eV.

The time-dependent Ce_2_^+^ signal was obtained as a function of pump–probe delay from −0.4 to 3 ps for a probe pulse centered at 408 nm and pump pulses at four different wavelengths: 1450, 1640, 1740, and 1860 nm, shown in Figures 3 and S9. The polarization of the probe pulse was set to the magic angle (θ = 55°) with respect to that of the pump pulse, to avoid rotational dynamics. For all four pump wavelengths, the transient signal (Figure S9) remains consistently low at negative delay times without clear oscillatory features, indicating inefficient Ce_2_^+^ formation. However, a significant rise in the transient is observed after time zero and accompanied by pronounced oscillatory modulations. The first maximum of the transient appears at a delay exceeding 200 fs, longer than the cross-correlation width of the two fs pulses, and therefore confirming the motion of the wave packet. The transients obtained for λ_pump_ = 1450 nm (0.86 eV) and λ_pump_ = 1640 nm (0.76 eV) show oscillatory features (Figure 3A,B), which are fitted using a cosine function with an exponential damping term (Figure S9 and Table S8). The prominent beating frequencies are determined to be 159 cm^–1^ and 199 cm^–1^, respectively. The transients obtained for λ_pump_ = 1740 nm (0.71 eV) and λ_pump_ = 1860 nm (0.67 eV) show additional high-frequency features (Figure 3C,D), involving the fitting frequencies of 233 cm^–1^ and 218 cm^–1^. We also measured the transients obtained by using a different polarization of the probe pulse (see Figure S10).

**Figure 3:**
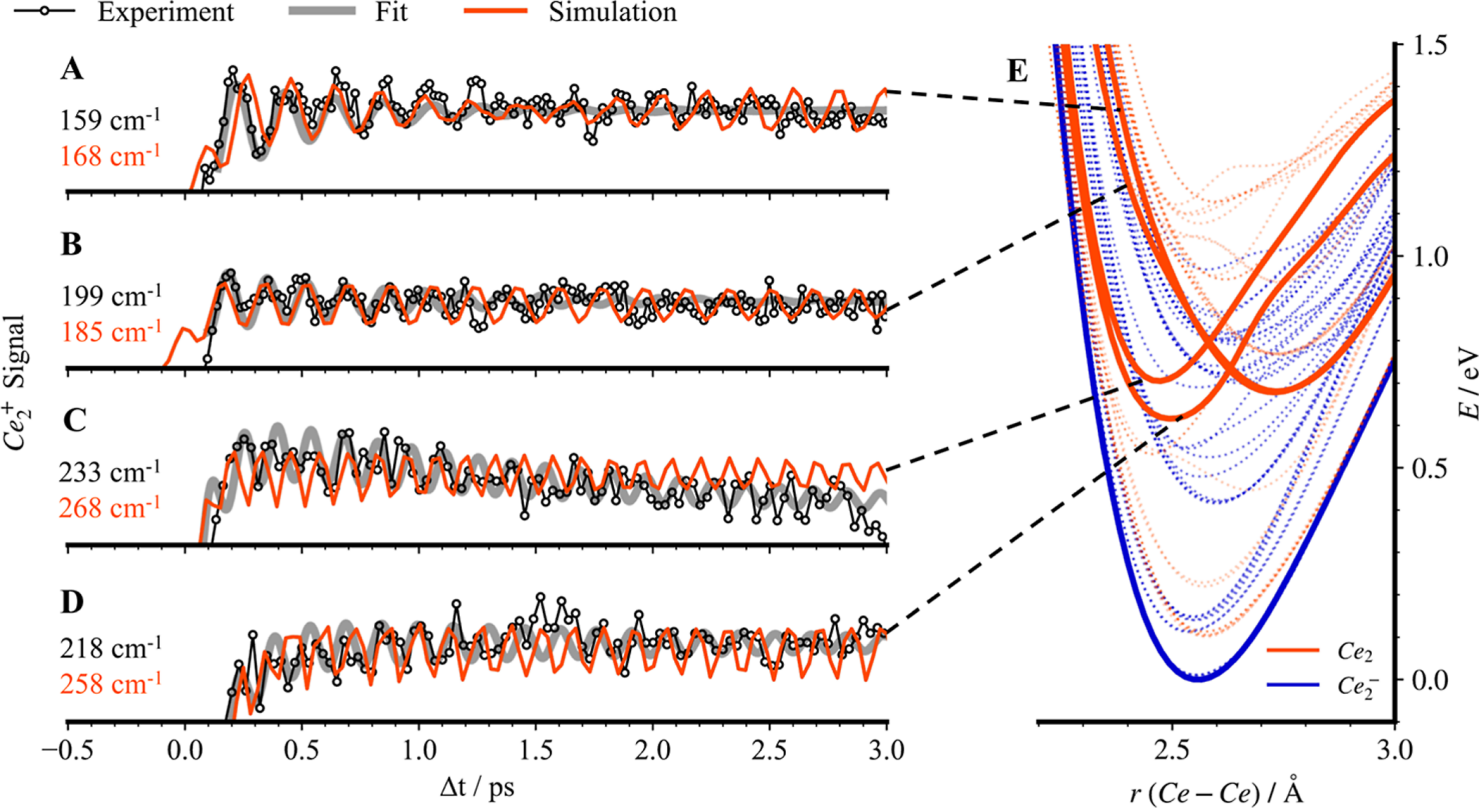
(A–D) Experimental and simulated Ce_2_^+^ transients over a delay time range from −1 to 3 ps with λ_probe_ = 408 nm. (A) λ_pump_ = 1450 nm (0.86 eV), (B) λ_pump_ = 1640 nm (0.76 eV), (C) λ_pump_ = 1740 nm (0.71 eV), and (D) λ_pump_ = 1860 nm (0.67 eV). Open dots (connected by black lines) represent the experimental data; red lines correspond to the simulations. The gray curves were obtained from a fit to the experimental results using a damped cosine function. See the Methods section in the Supporting Information for details. (E) Calculated PESs highlight the SO-coupled electronic states responsible for the quantum beats. Experimental beating frequencies (black) and simulated vibrational frequencies (red) at each wavelength (given in cm^–1^) correspond to (A) 1_u_, (B) 0_u_, (C) 4_g_, and (D) 0_g_^–^ SO states. Further information on the SO-coupled electronic states is provided in Tables S2–S4.

To understand the vibrational dynamics in the electronic states of Ce_2_ accessed after photodetachment, we performed full quantum dynamics simulations incorporating three charged states on precomputed PESs derived from SO-CASSCF calculations. The comparison of experiments and theory is given in Figure 3A–D and Table S5. The PESs (Figure 3E) highlight the Ce_2_ electronic states responsible for the observed vibrational wave-packet dynamics. The simulated oscillatory signals for λ_pump_ = 0.86 eV (Figure 3A) and λ_pump_ = 0.76 eV (Figure 3B) exhibit similar beating frequencies of 168 cm^–1^ and 185 cm^–1^, respectively, slightly higher than the experimental transients and supporting the theoretical assignment of the underlying wave-packet dynamics. A similar red shift of vibrational frequencies has been observed in previous studies of silver clusters,^37,38^ attributed to vibrational anharmonicity and the population of higher-lying vibrational states. The observed oscillations are associated with the 1_u_ and 0_u_ electronic states, located approximately 0.7 eV above the anion ground state. These states, which originate from the spin-pure ^3^Π_u_ electronic states (Table S3), result from the promotion of an electron from the bonding 5dπ_u_ molecular orbital (MO) of the Ce_2_ into the antibonding 6sσ_u_ orbital. The agreement between experiment and theory here further suggests that the photoelectron detachment occurs primarily from the 5π_u_ bonding orbitals around 0.8 eV. The observed lower beating frequencies indicate that the Ce–Ce bond in these states is weaker than a typical Ce–Ce triple bond, consistent with the vibrational frequencies of the ground electronic state of Ce_2_.^26,27^

In contrast to the previous results, the transients obtained for λ_pump_ = 0.71 eV (Figure 3C) and λ_pump_ = 0.67 eV (Figure 3D) exhibit higher frequency features, with fitted frequencies of 233 cm^–1^ and 218 cm^–1^. The corresponding simulated oscillatory features show slightly less agreement with experiment, yielding beating frequencies of 268 cm^–1^ and 258 cm^–1^, respectively. These oscillations originate from vibrational wave-packet dynamics in the 4g and 0g^–^ states, which originate from the spin-pure ^1^Γ_g_ and ^3^Σ^–^ states (Table S2). These states are accessed by detaching an electron from the antibonding 6sσ_u_ orbital of Ce_2_^–^. Their valence structure preserves the (6sσ_g_)^2^(5dπ_u_)^4^ bonding framework, while the remaining two electrons are distributed in different orbitals that mimic 4f character within the chosen basis set. The higher oscillation frequencies observed in the experiment also indicate a stronger Ce–Ce bond, consistent with a triple-bond character. Although computational limitations prevent a complete assessment of the role of the two 4f electrons in such highly excited states, these findings confirm that our theoretical model is also able to appropriately capture the higher-lying electronic states of Ce_2_.

### The Role of 4f Electrons

To fully unravel the role of the two 4f electrons in Ce_2_^–^ and refine our calculated EA, we performed higher-level electronic structure calculations using extended multistate complete active space second-order perturbation theory (XMS-CASPT2) with an all-electron basis set, ensuring an accurate description of the 4f electrons. The PESs obtained from these calculations, shown in Figure 4A, reveal a dense network of low-lying electronic states, with 14 states identified for Ce_2_^–^ and six for Ce_2_, all within a narrow energy range of less than 0.35 eV. The spectroscopic constants derived for Ce_2_ agree well with previous studies (Table S7), and the absence of 4f involvement in bonding further supports the reliability of our calculations.^26,27^ Among the electronic states of Ce_2_^–^, the first set of identified electronic states, highlighted in dark blue in Figure 4A, includes the ^2^Σ_u_^–^ and ^2^6_g_ states as the lowest-lying ones. Five additional states (^2^Σ_g_^+^, ^2^Σ_g_^–^, ^2^Σ_u_^+^, ^4^6_g_, and ^4^Σ_g_^+^) lie only 0.05 eV higher. These states arise from an electron occupying the formally nonbonding 6sσ_u_ orbital, while the remaining two electrons reside in 4fϕ MOs, forming configurations of (4fϕ_u_)^2^, (4fϕ_u_)^1^(4fϕ_g_)^1^, and (4fϕ_g_)^2^.

**Figure 4:**
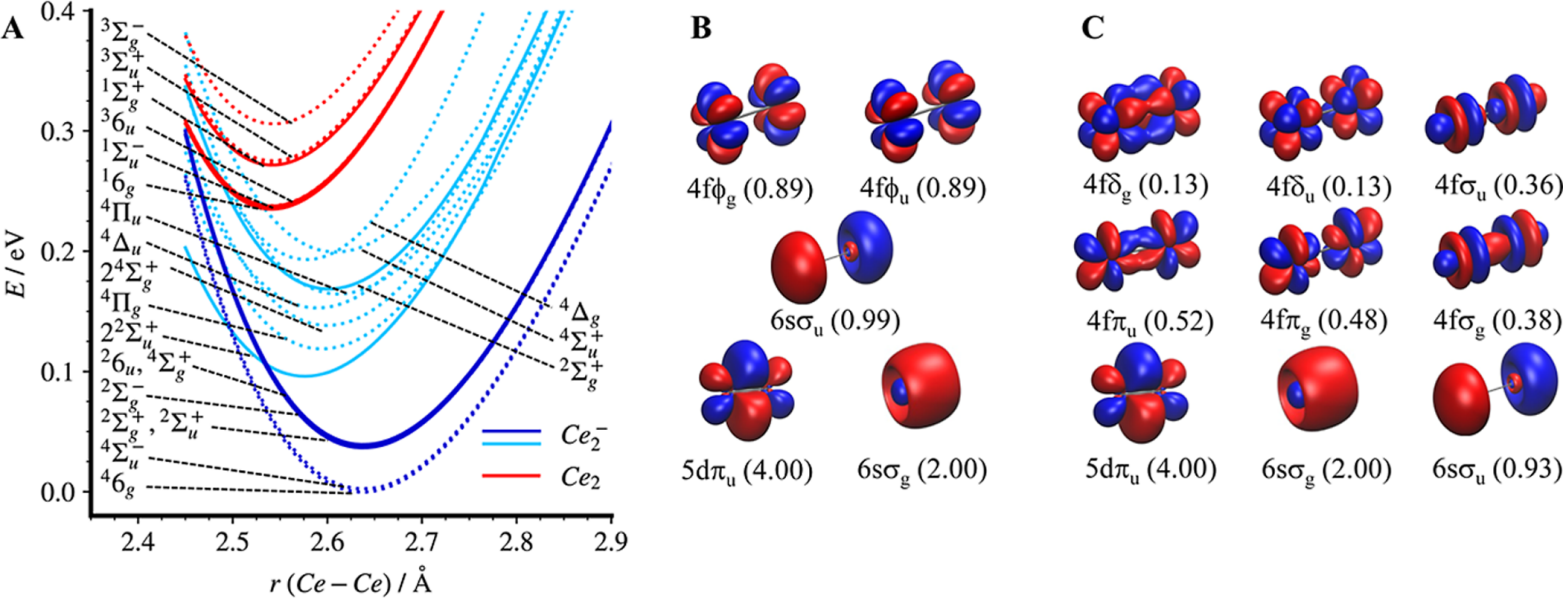
(A) Calculated PESs using SA-CASSCF/XMS-CASPT2 method. Solid curves represent singlet and doublet states, while dotted curves indicate triplet and quartet states. The dark blue and light blue curves correspond to the lowest-lying electronic states of Ce_2_^–^ and the excited states above 0.1 eV, respectively. Natural MOs and the occupation numbers of Ce_2_^–^ (in parentheses) are shown for (B) the dark blue states and (C) the light blue states. Note, the 6sσ_g_ and 5dπ_u_ orbitals are kept doubly occupied.

The calculated EA is 0.25 eV, which is higher than the previous SO-CASSCF value and in better agreement with the experimental EA, 0.24 eV. For the most stable electronic states of Ce_2_^–^, the calculation predicts a formal triple bond, with two electrons occupying the 4fϕ_g/u_ orbitals in the dominant configurations, resulting in an effective bond order of 2.5. The natural MOs contributing to bond formation are shown in Figure 4B, along with their occupations. One electron is localized in a nonbonding 6sσ_u_ orbital, reducing the bond order compared to Ce_2_, while the remaining two electrons mainly occupy atomic 4fϕ_g/u_ orbitals. Notably, the natural orbital occupations of the bonding (4fϕ_u_) and antibonding (4fϕ_g_) orbitals are identical, indicating that the two 4f electrons do not directly contribute to bond formation in the lowest-lying electronic states of Ce_2_^–^.

Beyond the lowest-energy electronic states, a second set of eight states, highlighted in light blue in Figure 4A, was identified only 0.1 eV higher in energy. Unlike the first set characterized by the occupation of the 4fϕ orbitals, these states involve alternative arrangements of the two 4f electrons, with increased occupation of the 4fσ, 4fπ, and 4fδ orbitals. Notably, this set features shorter equilibrium bond distances, ranging from 2.58 to 2.61 Å. Particularly, the natural MOs for these states (Figure 4C) reveal reduced occupation of the outer-shell antibonding 6sσ_u_ orbital, along with increased population of the bonding 4fσ_g_ and 4fπ_u_ orbitals. This redistribution of electron density suggests a direct covalent bonding contribution from the two 4f electrons, leading to an effective bond order of 2.6, higher than that of the first set. The calculated vibrational frequencies for these states range from 209 cm^–1^ to 212 cm^–1^, slightly higher than the 202 cm^–1^ to 207 cm^–1^ range found for the lowest-energy configurations (Table S6). This predicted increase in bond order, combined with the shorter equilibrium bond distance agrees well with the increase in the vibrational frequency. Here, covalent 4f-electron participation in these low-lying electronically excited states results in a remarkably stronger Ce–Ce bond. Such enhancement of 4f-electron interactions in metal–metal bonding has been theoretically predicted in lanthanide dimers such as La_2_ (4f^0^), Ce_2_ (4f^2^), and Pr_2_ (4f^4^), using CASSCF.^26^ The present work demonstrates the first investigation of 4f-electron participation in a covalent lanthanide metal–metal bond in electronically excited states, using the higher-level and more computationally demanding CASPT2 approach.

Notably, low-lying excited states around 0.1 eV (≈800 cm^–1^) have recently been observed in transition metal clusters, arising from the unexpectedly high density of states.^49,50^ Such a high density of states is uncommon in this low-energy range for a diatomic molecule but is particularly pronounced in the cerium dimer due to the occupation of 4f orbitals. The contribution of 4f-electrons to the bonding in the dimer therefore needs to be considered at warmer conditions, such as room temperature, where these low-energy electronic states are thermally populated by ≈2%.

Subtle structural changes can profoundly reshape the electronic landscape. Coordination with donor–acceptor ligands, such as CO or bulky ligands, is known to manipulate the properties of the coordination center,^21,51^ in particular, it can significantly alter the electronic structures of lanthanides in complexes.^52–54^ Recent studies provide evidence of 4f-orbital contributions to the ligand–Ce bond through strong 4f/5d hybridization.^15^ Additionally, the coordination contracts metal–metal bond lengths, leading to unique bonding motifs, have been previously reported for 5f metal–metal bonds.^6,55,56^ Here, we propose a similar ligand-induced contraction strategy aimed at stabilizing cerium complexes with direct 4f–4f interactions through the coordination that enforces a shorter Ce–Ce bond length. Notably, a prior study on the dicerium carbonyl complex Ce_2_CO in a solid argon matrix reveals a asymmetrically bound CO in a side-on bonding motif Ce_2_[μ_2_(μ–CO)], rather than in a simple dative coordination Ce_2_ ← CO.^57^ This further emphasizes the reducing nature and suggests its potential as a strong reducing agent.

## Conclusions

We present a comprehensive spectroscopic investigation of Ce_2_^–^ and Ce_2_ in the gas phase, using photoelectron and ultrafast spectroscopy combined with high-level quantum chemistry calculations. The electron affinity of Ce_2_ is experimentally determined to be 0.24 eV, from which a dissociation energy of 2.21 eV is derived for Ce_2_^–^. The wave-packet dynamics upon photodetachment are studied and yield vibrational frequencies for electronically excited states of Ce_2_. The ground electronic state of Ce_2_^–^ exhibits a conventional metal–metal triple bond with minimal 4f contributions. However, evidence of 4f electron participation in metal–metal bonding is found for the low-energy excited electronic states only 0.1 eV higher. Our results elucidate the bonding nature in Ce_2_^–^ and challenge the long-held notion of the lack of 4f-electron contribution to simple metal–metal bonds. The presence of 4f-orbital covalency in the Ce–Ce bond highlights the covalent contribution of f-electron bonding in the excited electronic states which can be thermally accessible. Moreover, we propose a promising strategy for forming stable complexes that contain lanthanide–lanthanide multiple bonds, where such 4f-covalent bonding can be stabilized by geometric confinement, favoring the formation of shorter metal–metal distances.

## Supplementary Material



## Notes

Detailed methods, additional results including APE spectra, photodepletion spectra, fs transients, calculated potential energy surfaces and electronic configurations, as well as simulated spectra (PDF)

## ⊥

Department of Chemistry, State Key Laboratory of Porous Materials for Separation and Conversion, Shanghai Key Laboratory of Molecular Catalysis and Innovative Materials, Fudan University, Songhu Rd. 2005, 200438 Shanghai, China.
