# Supplementary material for: Cerium Dimer Anion and the Contribution of 4f Electrons to Lanthanide Metal–Metal Bonds
Source: J Am Chem Soc. 2025 Sep 25;147(42):37977–84. doi: 10.1021/jacs.5c07348 (PMC12550857; doi:10.1021/jacs.5c07348)
Supplement: Supplementary file 1 [file ja5c07348_si_001.pdf]

Supporting Information of 'Cerium Dimer Anion and the Contribution of 4f Electrons to Lanthanide Metal-Metal Bonds'

Jiaye Jin′,[*,†,§] Nikita Kavka′,[‡] Tatsuya Chiba′,[¶] Max Grellmann,[†] Shiying Wang,[¶] Marcel Jorewitz,[†] Kit H. Bowen,[*,¶] Roland Mitric,[*,‡] and Knut R. Asmis[*,†]

† *Wilhelm-Ostwald-Institut für Physikalische und Theoretische Chemie, Universität Leipzig, Linnéstr. 2, 04103, Leipzig, Germany*

‡ *Institut für Physikalische und Theoretische Chemie, Universität Würzburg, Emil-Fischer Str. 42, 97074, Würzburg, Germany*

¶ *Department of Chemistry, Johns Hopkins University, Baltimore, Maryland 21218, United States*

§ *Present address: Department of Chemistry, State Key Laboratory of Porous Materials for Separation and Conversion, Shanghai Key Laboratory of Molecular Catalysis and Innovative Materials, Fudan University, Songhu Rd. 2005, 200438 Shanghai, China.*

E-mail: jyjin@fudan.edu.cn; kbowen@jhu.edu; roland.mitric@uni-wuerzburg.de; knut.asmis@uni-leipzig.de

Contents

Methods **3**

Leipzig University experimental methods . . . 3

Cluster preparation . . . 3

Femtosecond pump-probe spectroscopy . . . 3

Photodepletion spectroscopy. . . . 4

Frequency analysis . . . 5

Johns Hopkins University experimental methods . . . 5

Theory . . . 6

Electronic structure calculations . . . 6

Simulation of spectra . . . 8

Dynamic simulations . . . 8

Anion photoelectron spectra **10**

Potential energy surfaces calculations **12**

Simulated photoelectron spectra **16**

Photodepletion of Ce_2^- **18**

Fs NeNePo transients **19**

Calculated configurations **21**

Fit parameters **27**

References **28**

Methods

Leipzig University experimental methods

Fs NeNePo transients were measured using a tandem mass spectrometer with an integrated, cryogenically cooled linear quadrupole ion trap and a Ti:sapphire femtosecond laser system, as described previously.[1]

Cluster preparation

Cerium cluster anions, Ce_n^-, were produced by aggregation using a cerium target (99.9%, Evochem Advanced Materials GmbH) as the anode of a DC magnetron sputtering source (TORUS, Kurt J. Lesker) kept at 130 K using a liquid nitrogen cryostat. The contaminated surface layer of the cerium target was initially cleaned before being transferred into the vacuum chamber. Subsequently, the cerium target was sputtered for several hours to further clean the surface in the ultrahigh vacuum chamber. The plasma was produced and stabilized by applying a continuous argon (99.999%, Air Liquide) and helium (99.999%, Air Liquide) gas flow (16 SCCM for argon and 9 SCCM for helium). The beam of neutral and ionic particles from the sputtering source expanded around 35 mm distance through a 7 mm diameter nozzle and subsequently through a 2 mm diameter skimmer, which was held at a sufficiently positive potential in order to repel cations. The anion beam was collimated in a helium-filled radio frequency (RF) decapole ion guide of a tandem mass spectrometer for mass-selection and for fs pump-probe spectroscopy.

Femtosecond pump-probe spectroscopy

Ce_2^- ions were mass-selected by a quadrupole mass-filter and continuously accumulated in a cryogenic linear quadrupole ion trap (filled with 0.2 mbar helium buffer gas) held at 20 K. Two fs laser pulses, referred to as the pump pulse and the probe pulse, were collinearly combined and propagated along the trap axis and focused near the center of the ion trap

through a 1.5 mm thick CaF_2 lens (f = +1000 mm). The first vertically polarized fs laser pulse, tunable in wavelength between 1450 - 2400 nm (0.9 - 0.51 eV), was generated using an ultrafast travelling-wave optical parametric amplifier (TOPAS Prime, Light Conversion), was applied to photodetach an electron from the cold anion and launched a vibrational wave packet of Ce_2. Four pump wavelengths centered at 1860 nm (0.67 eV), 1740 nm (0.71 eV), 1640 nm (0.76 eV) and 1450 nm (0.86 eV) were applied in the present experiment. The temporal evolution of the vibrational wave-packet dynamics of Ce_2 was then probed using a second ultrafast laser pulse (centered at 408 nm) by two-photon ionization. The probe pulse was obtained by frequency doubling of the 800 nm fundamental in a harmonic generation unit (S/T-HG, Spectra-Physics), sent through a prism-pair compressor to minimize the group-velocity dispersion, and was then delayed by a linear translation delay stage (DLS225, Newport). The polarization of the probe pulse was adjusted between parallel ($\theta = 0°$) and perpendicular ($\theta = 90°$) with respect to the polarization plane of the pump pulse using a MgF_2 $\lambda/2$ waveplate (EKSMA).

Photodepletion spectroscopy.

Photodepletion spectroscopy was applied to study electronic properties of Ce_2^- under the irradiation at different wavelengths, inspired by the methods for measuring the photodetachment cross section of OH^-.[2,3] Ce_2^- ions were continuously accumulated in a helium-filled linear quadrupole ion trap (0.20 mbar partial pressure). Collisions with the helium buffer gas thermalize anions close to the trap temperature held at 20 K. The unfocused laser pulse propagated along the ion-trap axis to maximize the overlap with the ion cloud. The trapped anions absorbed photons, leading to photodetachment of electrons. Every 1000 ms, all anions were extracted from the trap, analyzed by a quadrupole mass filter and detected by a picoammeter (Keithley Model 6485). Ce_2^- signals with laser irradiation (I_{on}) and without irradiation (I_{off}) were measured at different wavelengths, yielding the photodepletion spectrum of Ce_2^-. The relative photodepletion cross section $\sigma_{pd}(\nu)$ is determined as $\frac{-1}{\mathrm{P}(\nu)} \cdot ln\frac{I_{on}}{I_{off}}$,

where $P(\nu)$ was the photon flux determined by measuring the laser power behind the vacuum chamber.

Frequency analysis

The oscillatory part of transients is fitted to a cosine function with an exponential damping term, as $f(t) = Acos(2\pi tc\omega_e + \phi)e^{-t/\tau}$, where A (a.u.) is the oscillation amplitude, τ (ps) is the oscillatory life time, ω_e (cm^{-1}) is the oscillation frequency, c is the speed of light in a vacuum, and ϕ (π) is the initial phase of the oscillation.

Johns Hopkins University experimental methods

Anion photoelectron spectroscopy of Ce_2^- was performed with an apparatus consisting of a laser vaporization ion source, a time-of-flight mass spectrometer, a magnetic bottle electron energy analyzer, and a velocity map imaging (VMI) electron energy analyzer, described elsewhere.[4,5] Ce_2^- ions were produced by ablating a translating and rotating cerium rod with a third harmonic output of a Nd:YAG laser (Continuum, Surelite II-10, 355 nm) while helium buffer gas (100 psi) was pulsed over it almost simultaneously from a pulse valve (Parker-Hannifin). The formed anions were sent through a skimmer to a Wiley-McLaren type time-of-flight mass spectrometer. For magnetic bottle photoelectron spectroscopy,[4] Ce_2^- (m/z = 280) ions were mass-gated, decelerated, and photodetached by the first, second, and third harmonic outputs of a Nd:YAG laser (Continuum, Surelite I-20, 1064 nm, 532 nm, and 355 nm, respectively). The kinetic energy of the photodetached electrons was measured by a magnetic bottle electron energy analyzer (resolution $\approx$ 35 meV at 1 eV electron kinetic energy). For VMI photoelectron spectroscopy,[5] the anions were mass-gated and photodetached by the linearly polarized first harmonic output of the Nd:YAG laser (1064 nm). The photodetached electrons were extracted toward a position-sensitive pulsed MCP with a phosphor screen along the axis of the ion beam, and the phosphor screen was recorded by a CCD camera. The three-dimensional distribution of the electrons was

reconstructed from the raw two-dimensional image by the BASEX method.[6] The kinetic energy of the electrons was determined by the distance from the center of the reconstructed image (resolution $\Delta E/E \approx 0.03$). For both magnetic bottle and VMI photoelectron spectra, the binding energy profiles were obtained by the energy conservation relationship: $h\nu$ = EBE + EKE, where $h\nu$ is the photon energy, EBE is the electron binding energy, and EKE is the kinetic energy of the photodetached electron. The photoelectron spectra were calibrated by the well-known transitions of NO^-,[7] Pb^-,[8] and Cu^-[9] for 1064 nm, 532 nm, and 355 nm photons, respectively.

Theory

All presented results, obtained through the application of multiconfigurational quantum chemical methods, were performed using the MOLPRO quantum chemistry package.[10]

Electronic structure calculations

The electronic structure calculations for ${Ce_2}^-$, Ce_2, and ${Ce_2}^+$, employed for subsequent quantum dynamical calculations, were performed using the state-averaged (SA) CASSCF (Complete active space Self-Consistent Field). A 12-electron quasirelativistic 4f-in-core pseudopotential (MWB46), specially designed for treating tetravalent lanthanides by Hülsen et al.,[11] was employed alongside the contracted atomic basis set (7s6p5d2f1g)/[6s5p4d2f1g] of VQZ quality. The selection of this basis set was primarily motivated by the successful outcomes reported by Cao and Dolg,[12] who utilized the large core ECP in their study of Ce_2, as well as by findings from Roos et al.,[13] which indicated that the 4f electrons maintain atomic character and have minimal impact on bonding. The orbital guess for the CASSCF calculations was generated from atomic densities. Dynamic correlation effects were omitted in the calculation due to technical challenges encountered during the computation of the potential energy surfaces (PESs). Spin-orbit (SO) coupling effects were introduced by diagonalizing the SO Hamiltonian in the basis of CASSCF wavefunctions. In order to avoid symmetry

breaking, all calculations were performed within the D_{2h} symmetry group. The active space included 15 orbitals derived from the 6s and 5d atomic orbitals with 8 active electrons for Ce_2, 7 for ${Ce_2}^+$, and 9 for ${Ce_2}^-$. The 5s and 5p core molecular orbitals were kept doubly occupied and subjected to optimization. State-averaging procedure included 16 singlet and 16 triplet states for Ce_2, and 16 doublet and 16 quartet states for ${Ce_2}^-$ and ${Ce_2}^+$. For all three species the PESs for all electronic states were calculated on a grid of 128 points by systematically varying the bond length between 1.75 and 10.25 Å. Cubic spline interpolation was employed to achieve a continuous representation of the PESs, resulting in a higher grid resolution of 400 points.

Additional electronic structure calculations were carried out for Ce_2 and ${Ce_2}^-$ utilizing the SA-CASSCF method, followed by extended multistate complete active space second-order perturbation theory (XMS-CASPT2) calculations to account for dynamic correlation effects. An all electron atomic natural orbital with relativistic core correlation, utilizing the triple-ζ plus polarization quality (ANO-RCC-VTZP) [9s8p6d4f3g2h] basis set, was employed. Scalar relativistic effects were addressed using the third-order Douglas-Kroll-Hess (DKH) Hamiltonian. The active space was determined by retaining the $(6s\sigma_g)^2(5d\pi_u)^4$ valence subconfiguration doubly occupied and including all other molecular orbitals derived from the 6s, 5d, and 4f atomic orbitals. This resulted in an active space of 2 and 3 electrons distributed across 23 orbitals for Ce_2 and ${Ce_2}^-$, respectively. Cao and Dolg employed a similar approach, maintaining this subconfiguration doubly occupied, in their investigation of Ce_2.[12] The state-averaging procedure included a total of 16 doublet and 16 quartet electronic states. The molecular orbitals formed by 5s and 5p atomic orbitals were included in the correlation treatment at the XMS-CASPT2 level of theory. To determine the dissociation energy of ${Ce_2}^-$, a total of 32 electronic states were computed for the Ce and Ce^- atoms at the CASSCF/XMS-CASPT2 level of theory. The corresponding active spaces comprised one 6s, three 6p, five 5d, and seven 4f orbitals, resulting in the distribution of 4 and 5 electrons across 16 orbitals for Ce and Ce^-, respectively. For atom calculations, the D_2 symmetry

group was utilized. The 5s and 5p semi-core electrons were included in the correlation treatment at the XMS-CASPT2 level of theory. All XMS-CASPT2 calculations employed a default electron affinity[14] of 0.25 and a level shift of 0.3. Potential energy surfaces were generated by calculating electronic states energies while varying the bond length near the equilibrium geometry within the range of 2.5 to 2.9 Å. Cubic spline interpolation was used to create a continuous representation of the PESs at a higher grid resolution of 400 points.

Simulation of spectra

In order to simulate the photoelectron spectrum of Ce_2^-, the vibrational eigenstates of each adiabatic electronic state of Ce_2 and Ce_2^- are calculated numerically on a grid of 400 points. The simulation starts with a Boltzmann distribution of the initial vibrational eigenstates of the lowest electronic states of Ce_2^- at different temperatures. Spectral line intensities were calculated using Franck-Condon factors obtained from the numerical overlap integrals between vibronic wavefunctions of the initial anion states (Ce_2^-) and final neutral states (Ce_2), without explicit evaluation of electronic transition dipole moments. The intensity for each transition was computed as

$$I_{i\to f} = \frac{2m_e}{\hbar^2}(e_f - e_i)\left|\langle\psi_f|\psi_i\rangle\right|^2, \qquad (1)$$

where e_i and e_f are the vibrational energies of the initial and final states, respectively, and ψ_i and ψ_f are their corresponding wavefunctions. The resulting stick spectrum was then convoluted with a Lorentzian profile to simulate the experimentally observed line broadening.

Dynamic simulations

The fs NeNePo transients are simulated through state-selected quantum dynamical calculations using pre-calculated SO-CASSCF PESs. The wave packet is initialized based on the Boltzmann distribution of the initial vibrational states in the lowest-lying electronic state

of Ce_2^- at 100 K, with vibrational eigenstates determined numerically on a grid using the Fourier representation of the kinetic energy operator. The wave packet is coupled with a Gaussian-shaped pump pulse, promoting it to a specific electronic state of Ce_2, with the dipole coupling strength set at a constant value. The wave packet is then propagated through the neutral electronic state and further excited to the lowest-lying Ce_2^+ state at different delay times using a Gaussian-shaped probe pulse. The propagation of the system is performed using the Split operator method. The resulting wave packet population in the Ce_2^+ electronic state is obtained for various time delays (Δt), which gives rise to the observed oscillatory signal. The dipole coupling strength between these electronic states is set to 1 a.u., the temporal widths of the pump and probe pulses, as well as the time step, are kept consistent with the parameters used in the experimental setup.

Anion photoelectron spectra

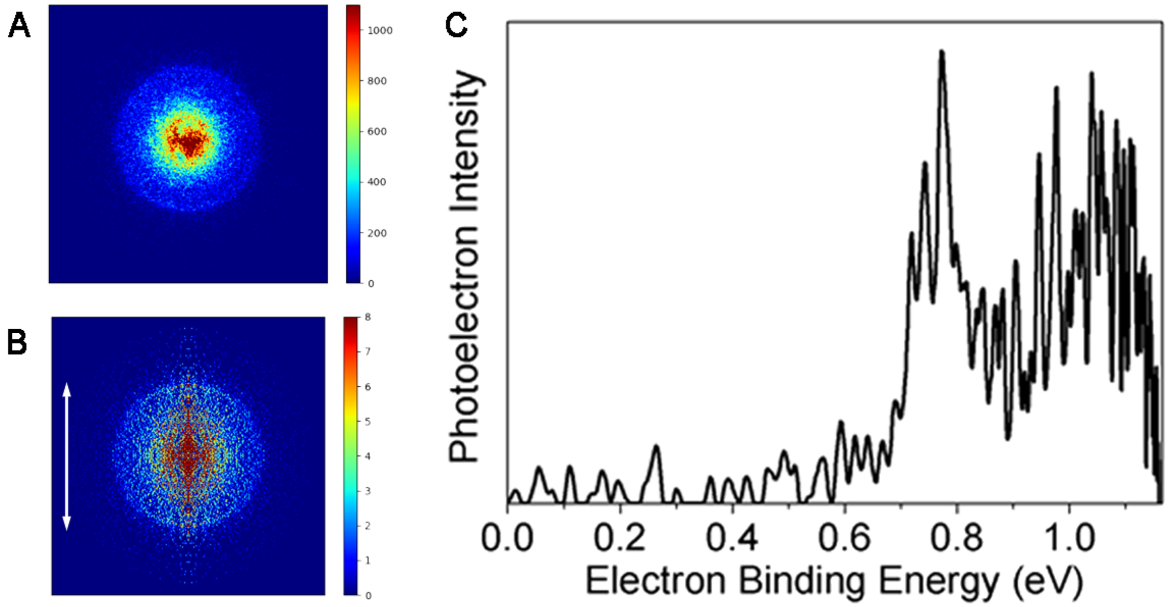

Figure S1: **VMI images and the APE spectrum.** (**A**) Raw photoelectron image of Ce_2^- taken by 1.17 eV (1064 nm) photon. The photodetachment laser is linearly polarized in the vertical direction. (**B**) Photoelectron image after reconstruction. The arrow in (**B**) indicates the laser polarization. (**C**) the APE spectrum obtained from VMI for the photoelectron of Ce_2^-.

.

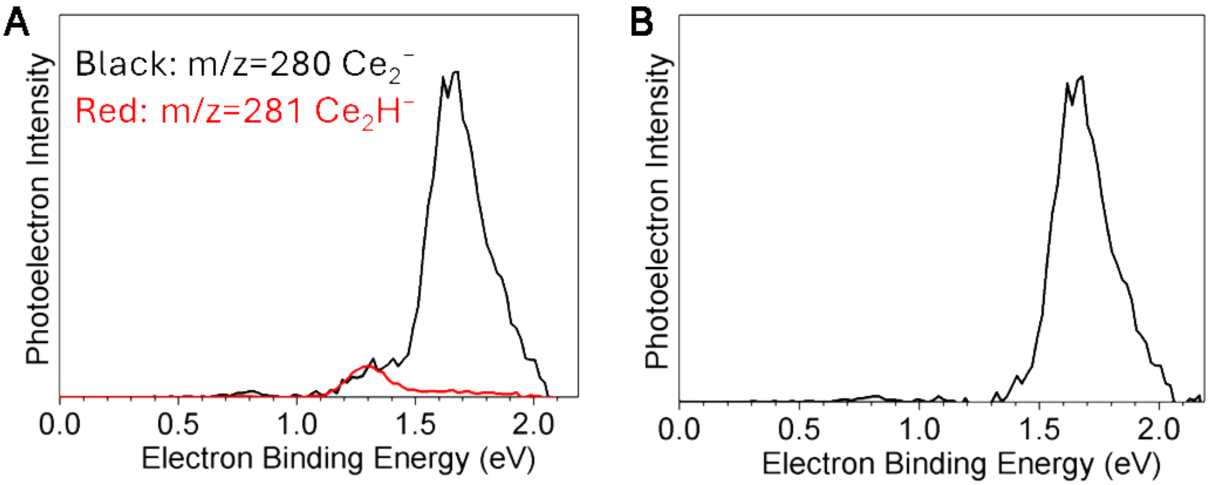

Figure S2: **APE spectra recorded at a photon energy of 2.33 eV.** (**A**) APE spectra of Ce_2^- (black) and Ce_2H^- (red) taken by 2.33 eV (532 nm) photon using a MB electron energy analyzer. A feature of an adjacent Ce_2H^- anion showed up in the spectrum of Ce_2^- at a weak intensity. (**B**) the APE spectrum of Ce_2^- obtained by subtracting the Ce_2H^- feature (red) from origin Ce_2^- spectrum (black) in (**A**).

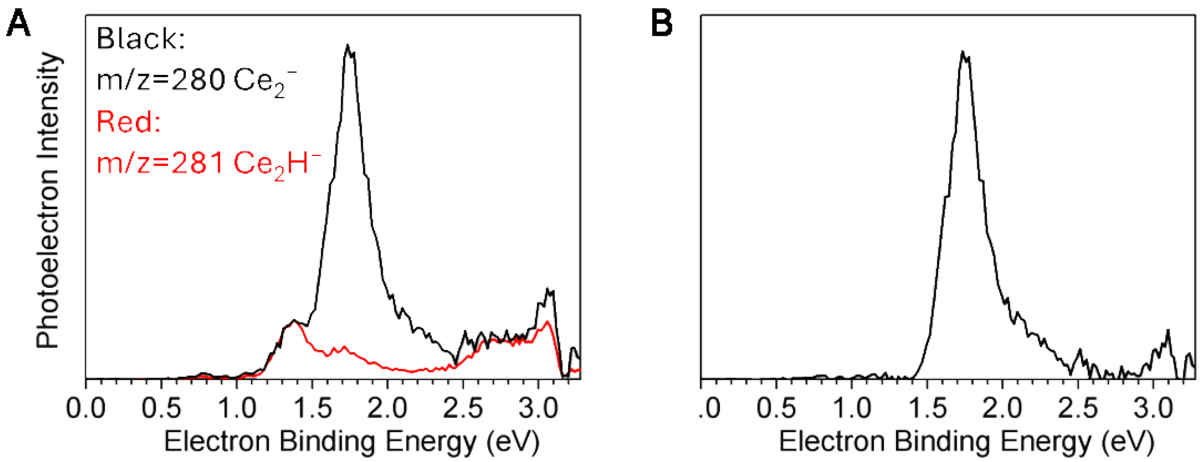

Figure S3: **APE spectra recorded at a photon energy of 3.49 eV.** (**A**) APE spectra of Ce_2^- (black) and Ce_2H^- (red) taken by 3.49 eV (355 nm) photon using a MB electron energy analyzer. Features of an adjacent Ce_2H^- anion showed up in the spectrum of Ce_2^- at a weak intensity. (**B**) the APE spectrum of Ce_2^- obtained by subtracting the Ce_2H^- feature (red) from origin Ce_2^- spectrum (black) in (**A**).

Potential energy surfaces calculations

The calculated CASSCF(8,15) PESs of Ce_2^+ , Ce_2 and Ce_2^- are provided in Figure S4. The figure illustrates the 12 lowest electronic states for each species. Upon initial inspection, the PESs reveal a highly complex structure, with closely spaced electronic states. Notably, a significant energy overlap between anionic and neutral states is observed, suggesting the plausible occurrence of vibrationally induced autoionization processes in this system.

Focusing on the neutral species, the calculation predicts the $^3\Delta_g$ electronic state as the ground state, with a marginal energy difference of only 0.1 eV to the next $^1\Delta_g$ electronic state, making it problematic to definitively determine the ground state of Ce_2. The dissociation energy is calculated to be 2.80 eV and 2.68 eV for $^3\Delta_g$ and $^1\Delta_g$, respectively, slightly exceeding the experimental value of 2.57 eV,[15] while also aligning well with theoretical results obtained using higher levels of theory.[13] A minimal ionization potential is estimated to be 4.15 eV and 4.17 eV for these states, respectively, which is lower than the experimental value of 4.9 eV.[16] The calculation yielded $^2\Delta_g$ electronic state as a ground state of Ce_2^+ with a bond order of 2. The lowest-lying state of the Ce_2^- is identified to be $^4\Delta_u$, closely followed by the $^2\Delta_g$ state, which is only 0.05 eV higher in energy. The electron affinity of the neutral Ce_2 is calculated to be 0.11 eV. Both electronic states exhibit a similar bonding pattern, forming a triple bond with six electrons in the bonding 6sσ_g and 5dπ_u orbitals, while the remaining two electrons are distributed in 5dσ_g and 5dδ_g orbitals. Tables S2-S4 provide spectroscopic constants for the lowest electronic states for Ce_2. Notably, the similarities in spectroscopic constants among electronic states with the same valence subconfiguration, including the discussed states, align well with the superconfigurational model proposed by Field.[17]

Figure S5 depicts the PESs of the three species considering SO-coupling. The visualization includes all calculated electronic states. The application of the SO coupling procedure resulted in a total of 64 electronic states for Ce_2 and 96 electronic states for both Ce_2^- and Ce_2^+ . The inclusion of SO-coupling results in only small changes to the PESs, causing mild

state splitting in specific electronic states. The alterations in computed spectroscopic constants are also relatively modest, consistent with findings in the work of Roos et al.[13] (See Table 2 in SI for details). The most notable changes occur in the energy range of 0.7 - 1.2 eV for Ce_2, where electronic states experience a noticeable shift towards closer energy spacing. Two singlet electronic states derived from $^1\Sigma_g^+$ symmetry are mixed with the triplet state $^3\Sigma_g^-$, leading to the emergence of double minima, a feature absent in the previous calculation. Notably, the ground states of all three species undergo no changes Details are given in Table S2.

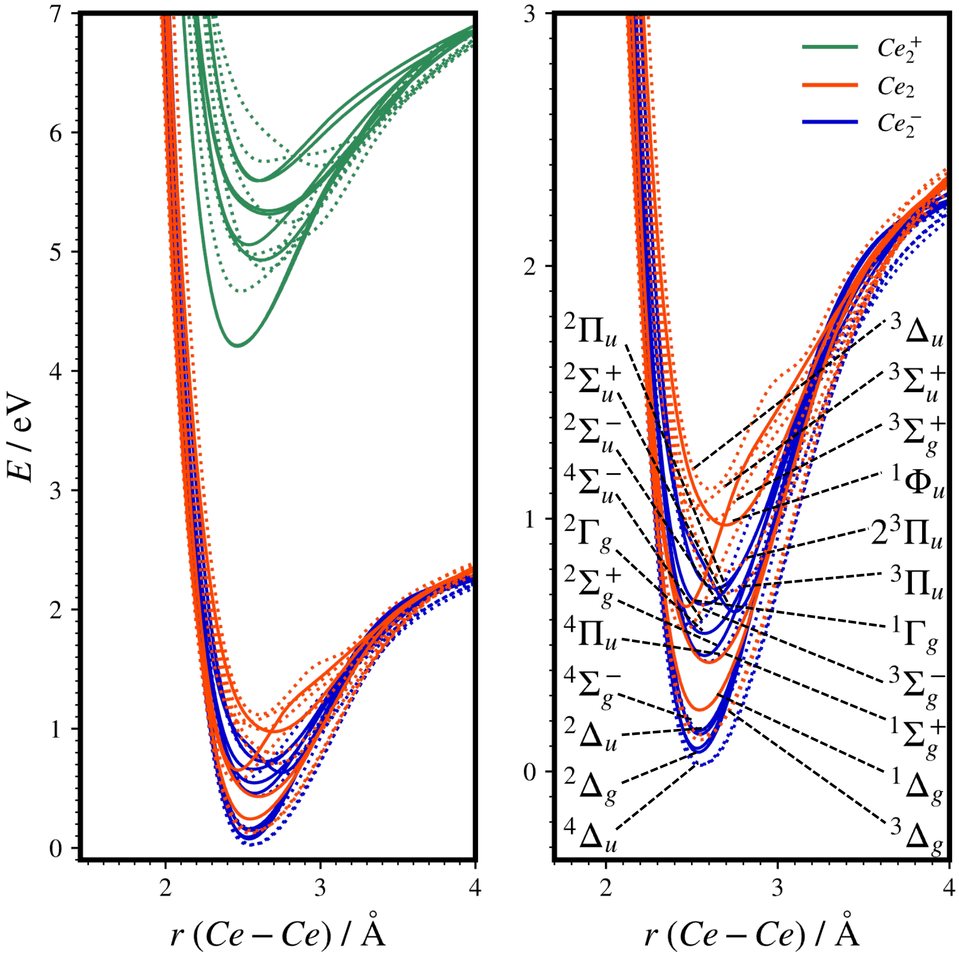

Figure S4: **Calculated spin-pure PESs for Ce_2, $Ce_2{}^+$, and $Ce_2{}^-$ using CASSCF method.** Left: An overview of the entire energy range displaying PESs for all three species. Right: A magnified view of Ce_2 and $Ce_2{}^-$ PESs with symmetry classification. Solid curves indicate singlet and doublet states, while dotted curves depict triplet and quartet states.

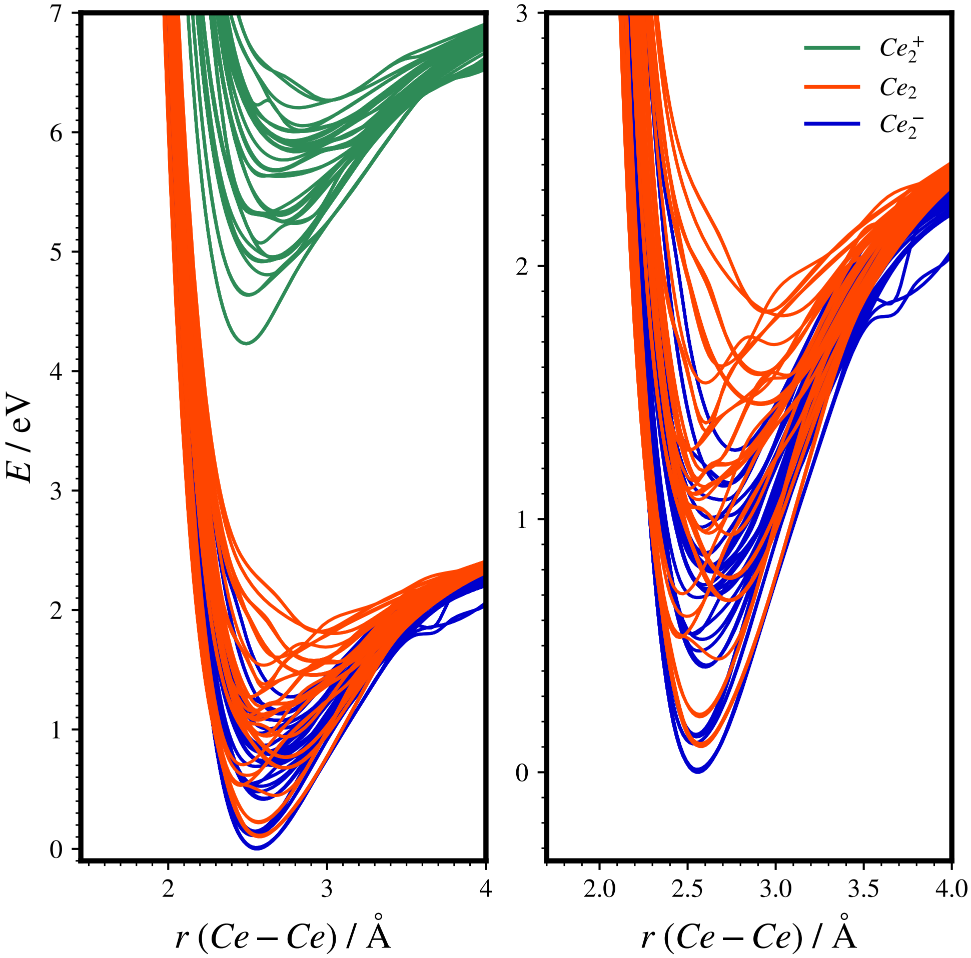

Figure S5: **Calculated SO-coupled PESs for Ce_2, $Ce_2{}^+$, and $Ce_2{}^-$ using SO-CASSCF.** Left: An overview of the entire energy range displaying PESs for all three species. Right: A magnified view of Ce_2 and $Ce_2{}^-$ PESs.

Simulated photoelectron spectra

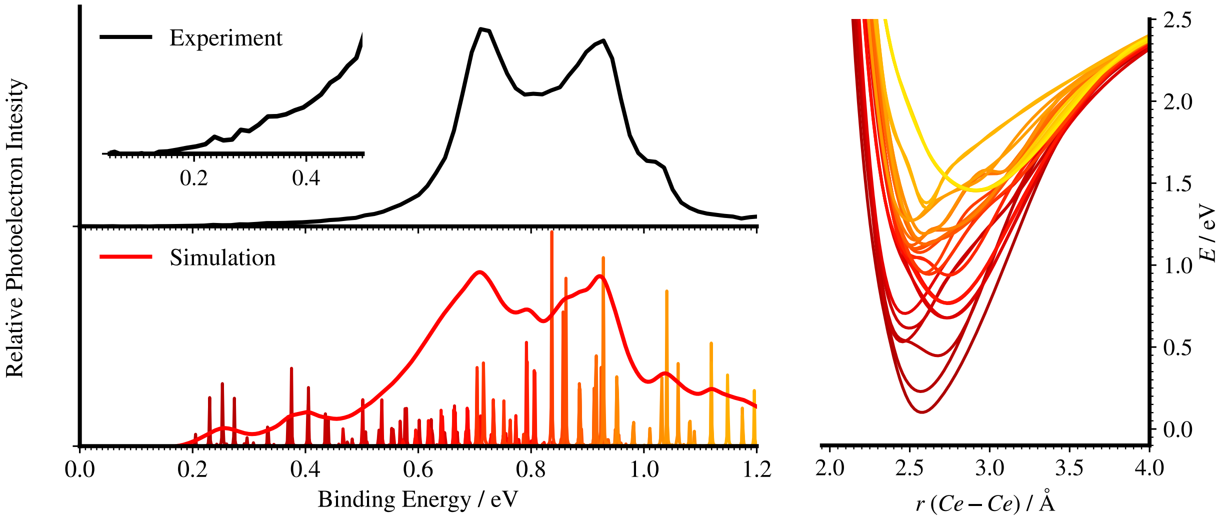

Figure S6: **Comparison of the experimental APE spectrum with the simulation at 50 K.** The calculated spectra are shifted by −0.24 eV and folded with a Lorentzian line profile with 0.055 eV FWHM. The red-yellow color gradient of the sticks represents the corresponding PES, with the gradient indicating ascending energy values. See Figure S7 for the simulations at different temperatures.

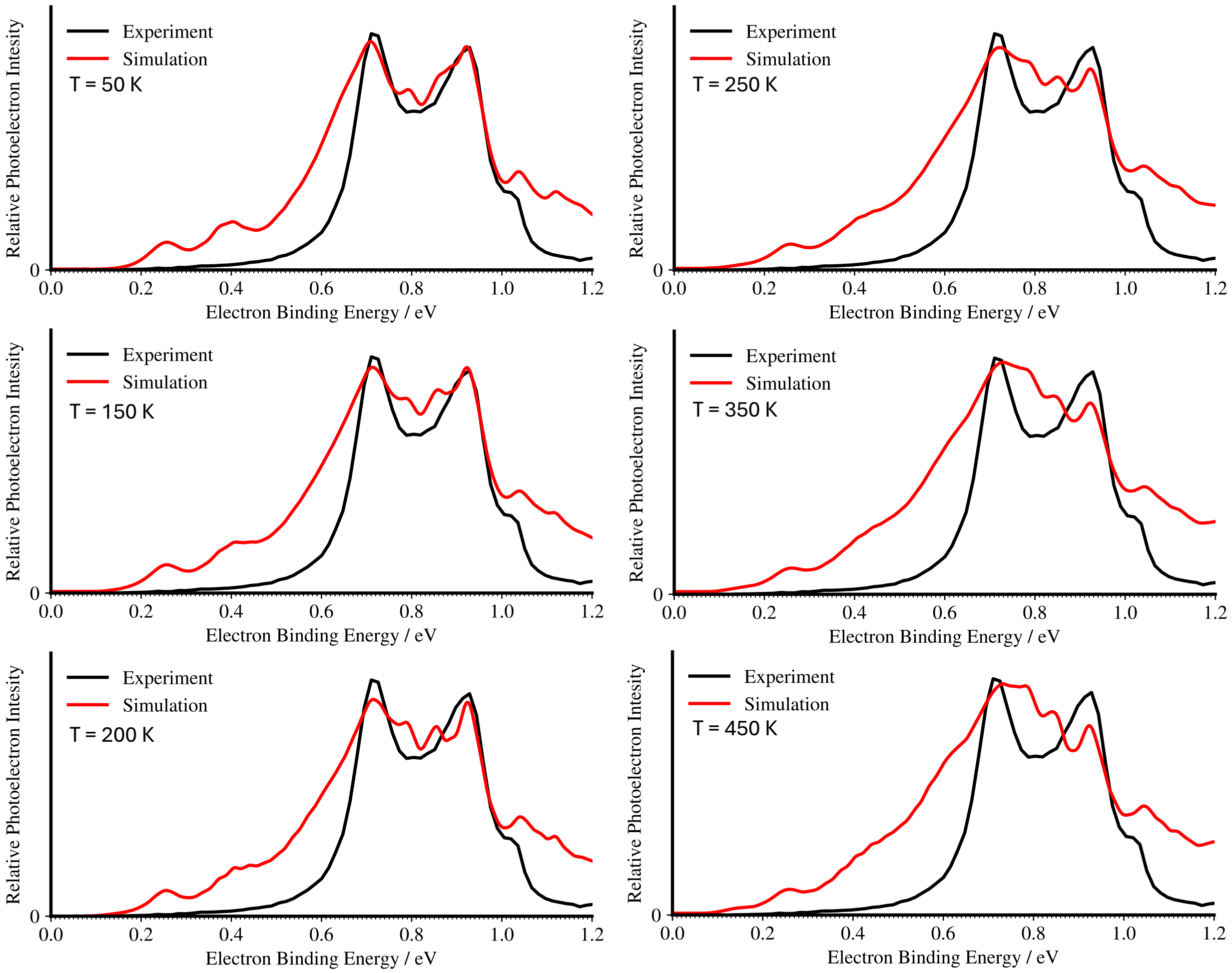

Figure S7: **Simulated photoelectron spectra at different temperatures.** The simulated spectra are calculated at 50 K, 150 K, 200 K, 250 K, 350 K and 450 K. The calculated spectra are shifted by −0.24 eV and folded with a Lorentzian line profile with 0.055 eV FWHM.

Photodepletion of Ce_2^-

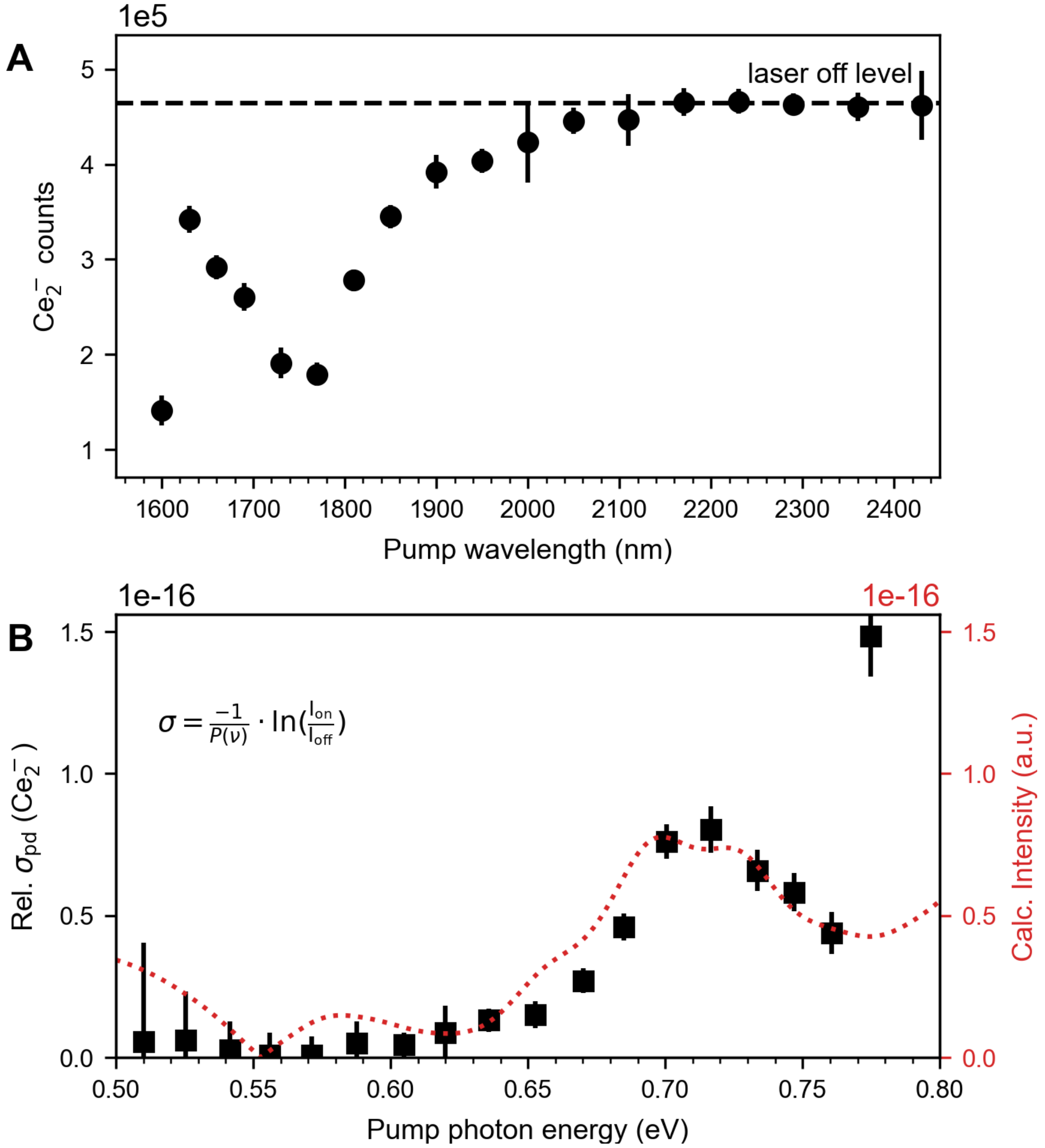

Figure S8: **Photodepletion of Ce_2^- yield in a cold ion trap and the relative photodepletion cross section** σ_{PD}**.** (**A**) The anion yield was obtained by filling in the ion trap for 1000 ms with Ce_2^- and under the irradiation at different wavelengths during the filling cycle. (**B**) The relative photodepletion cross section σ_{PD} of Ce_2^- as a function of photon energy from 0.5 eV to 0.8 eV. The red dotted line shows the convoluted spectrum using a Lorentzian line shape with 0.055 eV width from a Franck-Condon analysis of the transitions between Ce_2^- and Ce_2 obtained by CASSCF method. See Method section for details. The error bars in **A** and **B** represent the standard deviation obtained from independent measurements.

Fs NeNePo transients

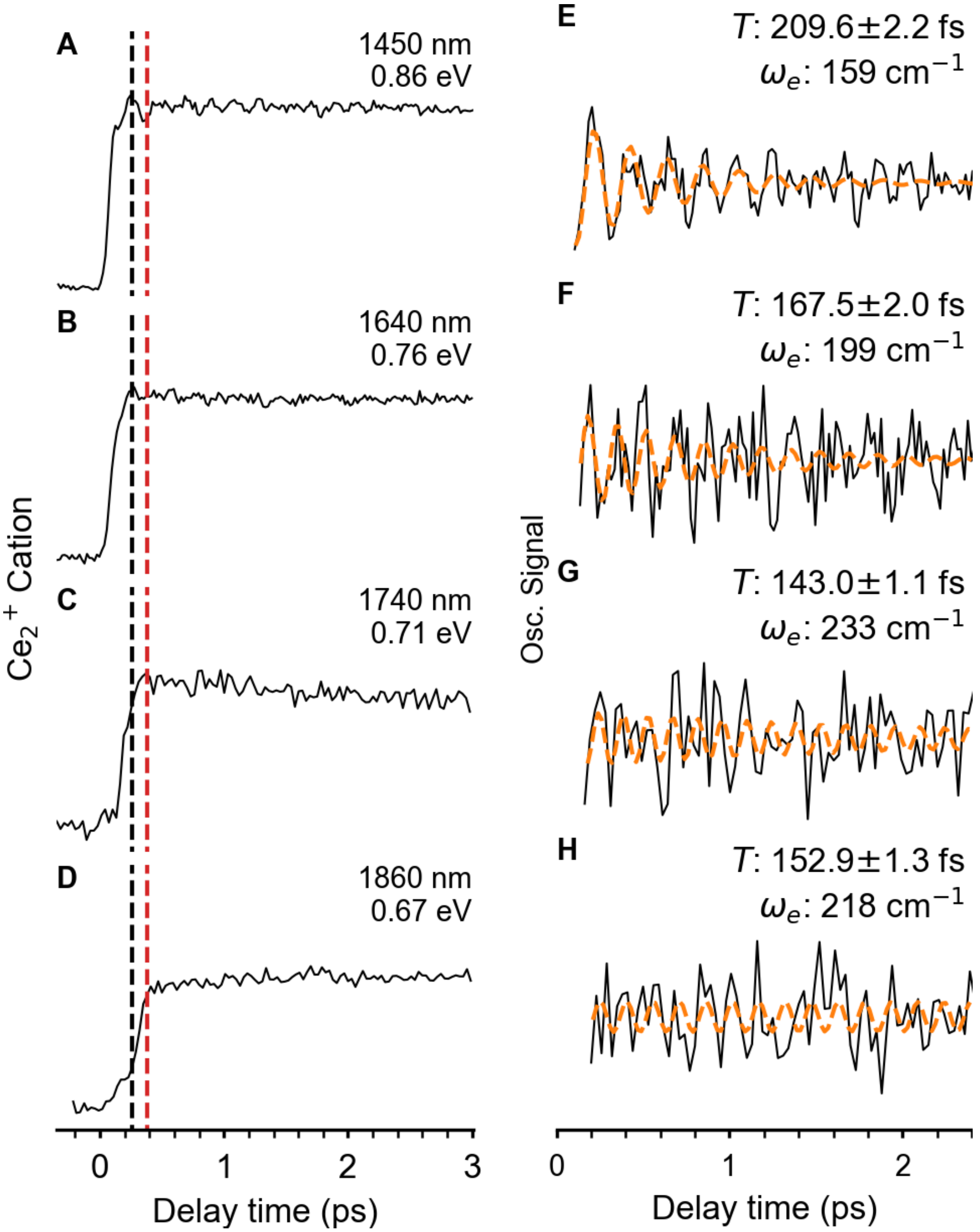

Figure S9: **Fs NeNePo transients probed at $\theta = 55°$.** Time-dependent Ce_2^+ signals (**A**-**D**) and the oscillatory components (**E**-**H**) for delay times from −0.2 to 3.0 ps. Four pump wavelengths λ_{pump} are applied. (**A**) and (**E**) for λ_{pump} = 1450 nm (0.86 eV); (**B**) and (**F**) for λ_{pump} = 1640 nm (0.76 eV); (**C**) and (**G**) for λ_{pump} = 1740 nm (0.71 eV) and (**D**) and (**H**) for λ_{pump} = 1860 nm (0.67 eV). λ_{probe} = 408 nm (3.03 eV). The polarization of the probe pulse is set at $\theta = 55°$. Black and red dashed lines in (**A**)-(**D**) mark the delay times of different maxima in transients. A cosine function containing an exponential damping is assumed for fitting the oscillatory signal (shown in orange). See method section for details.

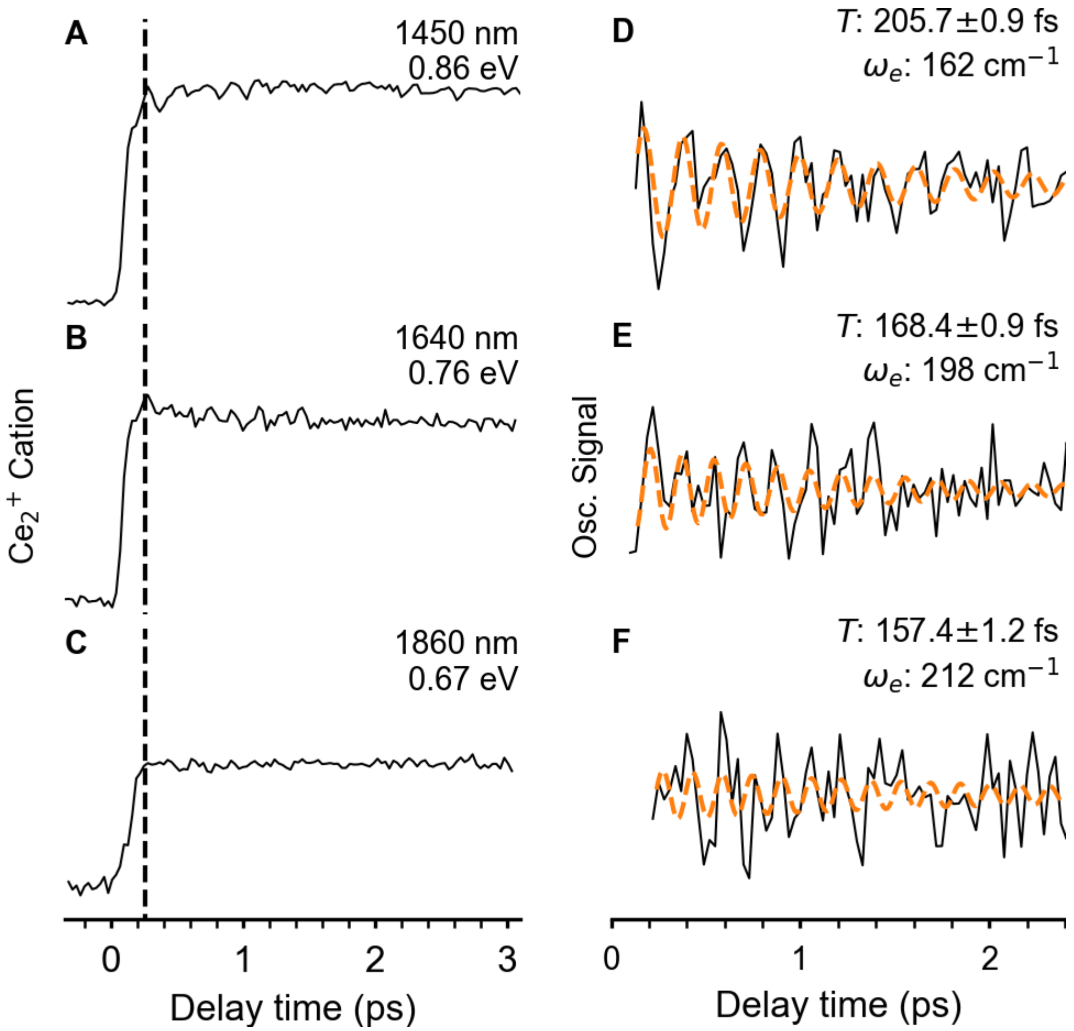

Figure S10: **Fs NeNePo transients probed at $\theta = 0°$.** Time-dependent Ce_2^+ signals (A-C) and the oscillatory components (D-F) for delay times from −0.2 to 3.0 ps. Three pump wavelengths λ_{pump} are applied. (**A**) and (**D**) for λ_{pump} = 1450 nm (0.86 eV); (**B**) and (**E**) for λ_{pump} = 1640 nm (0.76 eV), and (**C**) and (**F**) λ_{pump} = 1860 nm (0.67 eV). λ_{probe} = 408 nm (3.03 eV). The polarization of the probe pulse is set at $\theta = 0°$. Black and red dashed lines in (**A**)-(**C**) mark the delay time of the first maximum in transients. A cosine function containing an exponential damping is assumed for fitting the oscillatory signal (shown in orange). See method section for details.

Calculated configurations

Table S1: **Calculated electronic states using CASSCF method.** Predominant electronic configurations, relative adiabatic energies (ΔE, in eV), vertical excitation energies (VEE, in eV) from the lowest-energy electronic state and numerically calculated vibrational frequencies (ω_e, in cm^{-1}) of the electronic states of Ce_2^-, Ce_2 and Ce_2^+ obtained using CASSCF calculations.

	State	Predominant configuration	ΔE	VEE	ω_e
Ce_2^-	$^4\Delta_u$	$(5d\pi_u)^4(6s\sigma_g)^2(6s\sigma_u)^1(5d\sigma_g)^1(5d\delta_g)^1$	0.00	0.00	249
	$^2\Delta_g$	$(5d\pi_u)^4(6s\sigma_g)^2(5d\sigma_g)^2(5d\delta_g)^1$	0.06	0.08	259
	$^2\Delta_u$	$(5d\pi_u)^4(6s\sigma_g)^2(6s\sigma_u)^1(5d\sigma_g)^1(5d\delta_g)^1$	0.13	0.13	261
	$^4\Sigma_g^-$	$(5d\pi_u)^4(6s\sigma_g)^2(5d\sigma_g)^1(5d\delta_g)^2$	0.15	0.15	253
Ce_2	$^3\Delta_g$	$(5d\pi_u)^4(6s\sigma_g)^2(5d\sigma_g)^1(5d\delta_g)^1$	0.00	0.12	237
	$^1\Delta_g$	$(5d\pi_u)^4(6s\sigma_g)^2(5d\sigma_g)^1(5d\delta_g)^1$	0.10	0.19	251
	$^1\Sigma_g^+$	$(5d\pi_u)^4(6s\sigma_g)^2(5d\sigma_g)^2$	0.32	0.51	238
	$^3\Sigma_g^-$	$(5d\pi_u)^4(6s\sigma_g)^2(5d\delta_g)^1(5d\delta_g)^1$	0.45	0.63	311
	$^1\Gamma_g$	$(5d\pi_u)^4(6s\sigma_g)^2(5d\delta_g)^1(5d\delta_g)^1$	0.56	0.74	304
	$^3\Pi_u$	$(5d\pi_u)^3(6s\sigma_g)^2(5d\sigma_g)^1(5d\delta_g)^2$	0.54	0.84	239
	$2^3\Pi_u$	$(5d\pi_u)^3(6s\sigma_g)^2(5d\sigma_g)^2(5d\delta_g)^1$	0.65	0.91	197
	$^1\Phi_u$	$(5d\pi_u)^3(6s\sigma_g)^2(5d\sigma_g)^2(5d\delta_g)^1$	0.82	1.11	220
	$^3\Sigma_u^+$	$(5d\pi_u)^4(6s\sigma_g)^2(6s\sigma_u)^1(5d\sigma_g)^1$	0.91	1.04	214
	$^3\Delta_u$	$(5d\pi_u)^4(6s\sigma_g)^2(6s\sigma_u)^1(5d\delta_g)^1$	0.99	1.12	305
Ce_2^+	$^2\Delta_g$	$(5d\pi_u)^4(6s\sigma_g)^2(5d\delta_g)^1$	4.07	4.18	296

Table S2: **Calculated electronic states using SO-CASSCF method (Part 1).** Calculated relative energies of the SO-coupled electronic states using CASSCF method, along with the corresponding dominant spin-free precursor CASSCF states, with respect to the lowest-lying neutral (E(0)) and anionic (E(−)) states, equilibrium bond lengths (R_e), frequencies (ω_e), and dissociation energies (D_e) of the Ce_2 dimer (Part 1).

State number	SO state	Precursor state	$E(0)$ / eV	$E(-)$ / eV	R_e / Å	ω_e / cm^{-1}	D_e / eV
1	3_g	$^3\Delta_g$	0.00	0.10	2.58	253.77	2.81
2	2_g	$^3\Delta_g$	0.00	0.10	2.57	246.70	2.81
3	1_g	$^3\Delta_g$	0.00	0.10	2.58	249.09	2.81
4	2_g	$^3\Delta_g$	0.00	0.11	2.58	248.85	2.81
5	1_g	$^3\Delta_g$	0.01	0.11	2.57	234.66	2.81
6	3_g	$^3\Delta_g$	0.01	0.11	2.57	234.29	2.81
7	2_g	$^1\Delta_g$	0.12	0.22	2.57	249.31	2.70
8	2_g	$^1\Delta_g$	0.13	0.23	2.57	237.75	2.70
9	0_g^+	$^1\Sigma_g^+$	0.35	0.45	2.68	201.95	2.47
10	1_g	$^3\Sigma_g^-$	0.43	0.53	2.46	300.20	2.40
11	1_g	$^3\Sigma_g^-$	0.43	0.53	2.46	299.96	2.40
12	0_g^-	$^3\Sigma_g^-$	0.51	0.62	2.49	239.09	2.31
13	2_u	$^3\Pi_u$	0.58	0.68	2.73	193.47	2.23
14	1_u	$^3\Pi_u$	0.58	0.68	2.73	199.46	2.23
15	0_u	$^3\Pi_u$	0.58	0.68	2.73	193.48	2.25
16	0_u	$^3\Pi_u$	0.58	0.68	2.73	197.67	2.23
17	2_u	$^3\Pi_u$	0.58	0.68	2.73	197.68	2.23
18	1_u	$^3\Pi_u$	0.58	0.68	2.73	195.58	2.24
19	4_g	$^1\Gamma_g$	0.60	0.70	2.47	275.94	2.22

Table S3: **Calculated electronic states using SO-CASSCF method (Part 2).** Calculated relative energies of SO-coupled electronic states using CASSCF method, along with the corresponding dominant spin-free precursor CASSCF states, with respect to the lowest-lying neutral (E(0)) and anionic (E(−)) states, equilibrium bond lengths (R_e), frequencies (ω_e), and dissociation energies (D_e) of the Ce_2 dimer (Part 2).

State number	SO state	Precursor state	$E(0)$ / eV	$E(-)$ / eV	R_e / Å	ω_e / cm^{-1}	D_e / eV
20	1_u	$2^3\Pi_u$	0.66	0.77	2.73	197.50	2.15
21	0_u^-	$2^3\Pi_u$	0.67	0.77	2.73	190.22	2.15
22	0_u^-	$2^3\Pi_u$	0.67	0.77	2.73	193.33	2.16
23	2_u	$2^3\Pi_u$	0.67	0.77	2.73	190.65	2.16
24	1_u	$2^3\Pi_u$	0.67	0.77	2.73	193.52	2.15
25	2_u	$2^3\Pi_u$	0.67	0.77	2.73	187.82	2.16
26	1_u	$^3\Sigma_u^+$	0.84	0.94	2.75	219.12	1.98
27	1_u	$^3\Sigma_u^+$	0.84	0.94	2.73	213.33	1.98
28	1_g	$^3\Sigma_g^+$	0.84	0.94	2.62	237.39	1.99
29	1_g	$^3\Sigma_g^+$	0.84	0.94	2.62	237.36	1.99
30	0_g^+	$^3\Sigma_g^+$	0.85	0.95	2.60	287.29	1.98
31	0_u	$^3\Sigma_u^+$	0.98	1.08	2.57	236.72	1.85
32	1_u	$^3\Delta_u$	1.00	1.10	2.55	221.64	1.83
33	3_u	$^3\Delta_u$	1.00	1.10	2.53	222.12	1.83
34	2_u	$^3\Delta_u$	1.02	1.12	2.51	309.09	1.81
35	1_u	$^3\Delta_u$	1.03	1.13	2.60	208.14	1.80
36	3_u	$^3\Delta_u$	1.03	1.13	2.60	207.80	1.80
37	2_u	$^3\Delta_u$	1.05	1.15	2.53	280.13	1.78
38	3_u	$^1\Phi_u$	1.06	1.16	2.57	309.53	1.77
39	3_u	$^1\Phi_u$	1.06	1.16	2.57	304.10	1.77
40	1_u	$^1\Pi_u$	1.09	1.19	2.58	233.19	1.74
41	1_u	$^1\Pi_u$	1.09	1.19	2.58	233.31	1.74

Table S4: **Calculated electronic states using SO-CASSCF (Part 3).** Calculated relative energies of the SO-coupled electronic states using CASSCF method, along with the corresponding dominant spin-free precursor CASSCF states, with respect to the lowest-lying neutral (E(0)) and anionic (E(−)) states, equilibrium bond lengths (R_e), frequencies (ω_e), and dissociation energies (D_e) of the Ce_2 dimer (Part 3).

State number	SO state	Precursor state	$E(0)$ / eV	$E(-)$ / eV	R_e / Å	ω_e / cm^{-1}	D_e / eV
42	2_u	$^1\Delta_u$	1.17	1.27	2.51	236.76	1.68
43	2_u	$^1\Delta_u$	1.17	1.28	2.51	328.05	1.66
44	1_u	$2^3\Delta_u$	1.24	1.35	2.60	445.76	1.60
45	3_u	$2^3\Delta_u$	1.25	1.35	2.60	445.29	1.60
46	2_u	$2^3\Delta_u$	1.28	1.38	2.60	379.61	1.58
47	0_g	$^3\Pi_g$	1.35	1.45	2.92	133.22	1.48
48	0_g	$^3\Pi_g$	1.35	1.45	2.92	131.05	1.48
49	2_g	$^3\Pi_g$	1.35	1.45	2.90	136.87	1.50
50	2_g	$^3\Pi_g$	1.35	1.45	2.92	131.60	1.47
51	1_g	$^3\Pi_g$	1.36	1.46	2.92	129.23	1.47
52	1_g	$^3\Pi_g$	1.36	1.46	2.92	129.04	1.47
53	0_u^-	$^1\Sigma_u^-$	1.44	1.54	2.60	245.56	1.41
54	4_g	$^3\Phi_g$	1.47	1.57	2.90	164.76	1.39
55	2_g	$^3\Phi_g$	1.47	1.57	2.92	163.26	1.40
56	2_g	$^3\Phi_g$	1.47	1.57	2.90	162.41	1.38
57	4_g	$^3\Phi_g$	1.47	1.57	2.92	162.89	1.36
58	3_g	$^3\Phi_g$	1.48	1.58	2.92	158.48	1.37
59	3_g	$^3\Phi_g$	1.48	1.58	2.92	158.46	1.37
60	0_u^-	$^1\Sigma_u^+$	1.50	1.60	2.70	211.12	1.36
61	1_g	$^1\Pi_g$	1.70	1.80	3.05	120.39	1.16
62	1_g	$^1\Pi_g$	1.70	1.80	3.05	120.22	1.16
63	5_g	$^1\mathrm{H}_g$	1.72	1.82	2.95	179.62	1.14
64	5_g	$^1\mathrm{H}_g$	1.72	1.82	2.95	171.30	1.14

Table S5: **Comparison of experimental frequencies with simulations.** Comparison of experimental beating frequencies (ω_e) with simulated frequencies obtained from the quantum dynamics calculations on SO-CASSCF PESs, alongside the corresponding photon energy of the pump pulse (λ_{pump}) and identified electronic states (given in Tables S2-S4).

Simulation				Experiment	
State number	State	λ_{pump} / (eV)	ω_e / (cm^{-1})	λ_{pump} / (eV)	ω_e / (cm^{-1})
26,27	1_u	0.92	168	0.86	159
21,22	0_u	0.82	185	0.76	199
19	4_g	0.79	268	0.71	233
12	0_g^-	0.69	258	0.67	218

Table S6: **Calculated electronic states of $Ce_2{}^-$ using XMS-CASPT2 method.** Calculated spectroscopic constants, relative energies and dominant electronic configurations of the low-lying electronic states of $Ce_2{}^-$ using XMS-CASPT2 method.

State	Configuration	ΔE / eV	R_e / Å	ω_e / cm^{-1}	D_e / eV
$^4 6_g$	$(5d\pi_u)^4(6s\sigma_g)^2(6s\sigma_u)^1(4f\phi_u)^1(4f\phi_g)^1$	0.00	2.64	206.49	1.72
$^4\Sigma_u^-$	$(5d\pi_u)^4(6s\sigma_g)^2(6s\sigma_u)^1(4f\phi_u)^1(4f\phi_u)^1$<br>$(5d\pi_u)^4(6s\sigma_g)^2(6s\sigma_u)^1(4f\phi_g)^1(4f\phi_g)^1$	0.00	2.64	207.50	1.73
$^4\Sigma_g^+$	$(5d\pi_u)^4(6s\sigma_g)^2(6s\sigma_u)^1(4f\phi_u)^1(4f\phi_g)^1$	0.04	2.64	206.59	1.69
$^2\Sigma_g^+$	$(5d\pi_u)^4(6s\sigma_g)^2(6s\sigma_u)^1(4f\phi_u)^1(4f\phi_g)^1$	0.04	2.64	206.20	1.69
$^2\Sigma_g^-$	$(5d\pi_u)^4(6s\sigma_g)^2(6s\sigma_u)^1(4f\phi_u)^1(4f\phi_g)^1$	0.04	2.66	202.18	1.69
$^2\Sigma_u^+$	$(5d\pi_u)^4(6s\sigma_g)^2(6s\sigma_u)^1(4f\phi_u)^2$<br>$((5d\pi_u)^4(6s\sigma_g)^2(6s\sigma_u)^1(4f\phi_g)^2$	0.04	2.64	206.47	1.69
$^2 6_u$	$(5d\pi_u)^4(6s\sigma_g)^2(6s\sigma_u)^1(4f\phi_u)^1(4f\phi_u)^1$<br>$(5d\pi_u)^4(6s\sigma_g)^2(6s\sigma_u)^1(4f\phi_g)^1(4f\phi_g)^1$	0.04	2.64	206.39	1.69
$2^2\Sigma_u^+$	$(5d\pi_u)^4(6s\sigma_g)^2(6s\sigma_u)^1(4f\sigma_g)^2$<br>$(5d\pi_u)^4(6s\sigma_g)^2(6s\sigma_u)^1(4f\sigma_u)^2$	0.10	2.58	209.34	2.91
$^4\Pi_g$	$(5d\pi_u)^4(6s\sigma_g)^2(6s\sigma_u)^1(4f\sigma_g)^1(4f\pi_u)^1$<br>$(5d\pi_u)^4(6s\sigma_g)^2(6s\sigma_u)^1(4f\sigma_u)^1(4f\pi_g)^1$	0.12	2.59	209.45	1.61
$2^4\Sigma_g^+$	$(5d\pi_u)^4(6s\sigma_g)^2(6s\sigma_u)^1(4f\sigma_g)^1(4f\sigma_u)^1$	0.14	2.60	212.71	1.59
$^4\Delta_u$	$(5d\pi_u)^4(6s\sigma_g)^2(6s\sigma_u)^1(4f\sigma_g)^1(4f\delta_g)^1$<br>$(5d\pi_u)^4(6s\sigma_g)^2(6s\sigma_u)^1(4f\sigma_u)^1(4f\delta_u)^1$	0.16	2.59	207.02	1.57
$^4\Pi_u$	$(5d\pi_u)^4(6s\sigma_g)^2(6s\sigma_u)^1(4f\sigma_g)^1(4f\pi_g)^1$<br>$(5d\pi_u)^4(6s\sigma_g)^2(6s\sigma_u)^1(4f\sigma_u)^1(4f\pi_u)^1$	0.17	2.61	210.22	1.56
$^2\Sigma_g^+$	$(5d\pi_u)^4(6s\sigma_g)^2(6s\sigma_u)^1(4f\sigma_g)^1(4f\sigma_u)^1$	0.17	2.60	212.39	1.56
$^4\Delta_g$	$(5d\pi_u)^4(6s\sigma_g)^2(6s\sigma_u)^1(4f\sigma_g)^1(4f\delta_u)^1$<br>$(5d\pi_u)^4(6s\sigma_g)^2(6s\sigma_u)^1(4f\sigma_u)^1(4f\delta_g)^1$	0.20	2.61	210.06	1.53
$^4\Sigma_u^+$	$(5d\pi_u)^4(6s\sigma_g)^2(5d\sigma_g)^1(4f\sigma_g)^1(4f\sigma_u)^1$	0.20	2.58	238.31	1.53
$^2\Pi_g$	$(5d\pi_u)^4(6s\sigma_g)^2(6s\sigma_u)^1(4f\sigma_g)^1(4f\pi_u)^1$<br>$(5d\pi_u)^4(6s\sigma_g)^2(6s\sigma_u)^1(4f\sigma_u)^1(4f\pi_g)^1$	0.22	2.60	209.62	1.63
$^2\Pi_u$	$(5d\pi_u)^4(6s\sigma_g)^2(6s\sigma_u)^1(4f\sigma_g)^1(4f\pi_g)^1$<br>$(5d\pi_u)^4(6s\sigma_g)^2(6s\sigma_u)^1(4f\sigma_u)^1(4f\pi_u)^1$	0.27	2.61	209.17	1.46

Table S7: **Calculated electronic states of Ce_2 using XMS-CASPT2 method.** Calculated spectroscopic constants, relative energies (with repect to the ground electronic state of $Ce_2{}^-$) and electronic configurations of the low-lying electronic states of Ce_2 using XMS-CASPT2 method.

State	Configuration	ΔE / eV	R_e / Å	ω_e / cm^{-1}	D_e / eV
$^1 6_g$	$(5\mathrm{d}\pi_u)^4(6\mathrm{s}\sigma_g)^2(4\mathrm{f}\phi_u)^1(4\mathrm{f}\phi_u)^1$<br>$(5\mathrm{d}\pi_u)^4(6\mathrm{s}\sigma_g)^2(4\mathrm{f}\phi_g)^1(4\mathrm{f}\phi_g)^1$	0.24	2.54	237.51	1.82
$^1\Sigma_u^-$	$(5\mathrm{d}\pi_u)^4(6\mathrm{s}\sigma_g)^2(4\mathrm{f}\phi_u)^1(4\mathrm{f}\phi_g)^1$	0.24	2.54	237.03	1.82
$^3 6_u$	$(5\mathrm{d}\pi_u)^4(6\mathrm{s}\sigma_g)^2(4\mathrm{f}\phi_u)^1(4\mathrm{f}\phi_g)^1$	0.24	2.54	237.17	1.82
$^1\Sigma_g^+$	$(5\mathrm{d}\pi_u)^4(6\mathrm{s}\sigma_g)^2(4\mathrm{f}\phi_u)^2$	0.27	2.54	236.27	1.79
$^3\Sigma_u^+$	$(5\mathrm{d}\pi_u)^4(6\mathrm{s}\sigma_g)^2(4\mathrm{f}\phi_u)^1(4\mathrm{f}\phi_g)^1$	0.28	2.54	236.17	1.79
$^3\Sigma_g^-$	$(5\mathrm{d}\pi_u)^4(6\mathrm{s}\sigma_g)^2(4\mathrm{f}\phi_u)^1(4\mathrm{f}\phi_u)^1$	0.31	2.54	235.28	1.75

Fit parameters

Table S8: **Fit parameters of oscillatory signals.** Oscillatory signals, shown in Figure S9 and S10, were fitted by the function $f(t) = Acos(2\pi tc\omega_e + \phi)e^{-t/\tau}$.

	$\theta = 55°$				$\theta = 0°$			
λ_{pump} (eV)	ω_e (cm^{-1})	ϕ (π)	A (a.u.)	τ (ps)	ω_e (cm^{-1})	ϕ (π)	A (a.u.)	τ (ps)
0.86	159	-0.1	36	0.6	162	-0.2	23	1.3
0.76	199	-0.2	21	0.8	198	0.0	13	1.1
0.71	233	-1.4	5	2.1				
0.67	218	-1.3	7	2.2	212	-1	11	2.1

References

(1) Jin, J.; Grellmann, M.; Asmis, K. R. Nuclear quantum dynamics on the ground electronic state of neutral silver dimer 107 Ag 109 Ag probed by femtosecond NeNePo spectroscopy. *Phys. Chem. Chem. Phys.* **2023**, *25*, 24313–24320.

(2) Trippel, S.; Mikosch, J.; Berhane, R.; Otto, R.; Weidemüller, M.; Wester, R. Photodetachment of cold OH- in a multipole ion trap. *Phys. Rev. Lett.* **2006**, *97*, 193003.

(3) Hlavenka, P.; Otto, R.; Trippel, S.; Mikosch, J.; Weidemüller, M.; Wester, R. Absolute photodetachment cross section measurements of the O- and OH- anion. *J. Chem. Phys.* **2009**, *130*.

(4) Liu, G.; Ciborowski, S. M.; Zhu, Z.; Chen, Y.; Zhang, X.; Bowen, K. H. The metalloformate anions, M (CO 2)-, M= Ni, Pd, Pt, formed by electron-induced CO 2 activation. *Phys. Chem. Chem. Phys.* **2019**, *21*, 10955–10960.

(5) Liu, G.; Ciborowski, S. M.; Graham, J. D.; Buytendyk, A. M.; Bowen, K. H. Photoelectron spectroscopic study of dipole-bound and valence-bound nitromethane anions formed by Rydberg electron transfer. *J. Chem. Phys.* **2020**, *153*.

(6) Dribinski, V.; Ossadtchi, A.; Mandelshtam, V. A.; Reisler, H. Reconstruction of Abel-transformable images: The Gaussian basis-set expansion Abel transform method. *Rev. Sci. Instrum.* **2002**, *73*, 2634–2642.

(7) Travers, M. J.; Cowles, D. C.; Ellison, G. B. Reinvestigation of the electron affinities of O2 and NO. *Chem. Phys. Lett.* **1989**, *164*, 449–455.

(8) Feigerle, C.; Corderman, R.; Lineberger, W. Electron affinities of B, Al, Bi, and Pb. *J. Chem. Phys.* **1981**, *74*, 1513–1515.

(9) Ho, J.; Ervin, K. M.; Lineberger, W. Photoelectron spectroscopy of metal cluster anions: Cu- n, Ag- n, and Au- n. *J. Chem. Phys.* **1990**, *93*, 6987–7002.

(10) Werner, H.-J.; Knowles, P. J.; Knizia, G.; Manby, F. R.; Schütz, M. Molpro: a general-purpose quantum chemistry program package. *WIREs Comput. Mol. Sci.* **2012**, *2*, 242–253.

(11) Hülsen, M.; Weigand, A.; Dolg, M. Quasirelativistic energy-consistent 4f-in-core pseudopotentials for tetravalent lanthanide elements. *Theor. Chem. Account* **2009**, *122*, 23–29.

(12) Cao, X.; Dolg, M. Electronic structure of lanthanide dimers. *Mol. Phys.* **2003**, *101*, 1967–1976.

(13) Roos, B. O.; Lindh, R.; Malmqvist, P.-Å.; Veryazov, V.; Widmark, P.-O.; Borin, A. C. New relativistic atomic natural orbital basis sets for lanthanide atoms with applications to the Ce diatom and LuF3. *J. Phys. Chem. A* **2008**, *112*, 11431–11435.

(14) Ghigo, G.; Roos, B. O.; Åke Malmqvist, P. A modified definition of the zeroth-order Hamiltonian in multiconfigurational perturbation theory (CASPT2). *Chem. Phys. Lett.* **2004**, *396*, 142–149.

(15) Shen, X.; Fang, L.; Chen, X.; Lombardi, J. R. Absorption, excitation, and resonance Raman spectra of Ce 2, Pr 2, and Nd 2. *J. Chem. Phys.* **2000**, *113*, 2233–2237.

(16) Hardy, R. A.; Karayilan, A. M.; Metha, G. F. Investigating Charge Transfer Interactions in AuCe2O n Clusters Using Photoionization Efficiency Spectroscopy and Density Functional Theory. *J. Phys. Chem. A* **2019**, *123*, 10158–10168.

(17) Field, R. W. Diatomic Molecule Electronic Structure beyond Simple Molecular Constants. *Ber. Bunsenges. Phys. Chem.* **1982**, *86*, 771–779.
